# Development of Small-Molecule STING Activators for Cancer Immunotherapy

**DOI:** 10.3390/biomedicines10010033

**Published:** 2021-12-24

**Authors:** Hee Ra Jung, Seongman Jo, Min Jae Jeon, Hyelim Lee, Yeonjeong Chu, Jeehee Lee, Eunha Kim, Gyu Yong Song, Cheulhee Jung, Hyejin Kim, Sanghee Lee

**Affiliations:** 1 Creative Research Center for Brain Science, Brain Science Institute, Korea Institute of Science and Technology, Seoul 02792, Korea; 120044@kist.re.kr (H.R.J.); 120542@kist.re.kr (H.L.); duswjd1415@ajou.ac.kr (Y.C.); tany828@kist.re.kr (J.L.); 2 Department of Biotechnology, College of Life Sciences and Biotechnology, Korea University, Seoul 02841, Korea; damo363@korea.ac.kr; 3 Therapeutics & Biotechnology Division, Korea Research Institute of Chemical Technology, Daejeon 34114, Korea; jsm558@krict.re.kr (S.J.); bluej936@krict.re.kr (M.J.J.); 4 Department of Pharmacy, College of Pharmacy, Chungnam National University, Daejeon 34134, Korea; gysong@cnu.ac.kr; 5 Department of Medicinal Chemistry and Pharmacology, University of Science & Technology, Daejeon 34113, Korea; 6 Department of Pharmacy, College of Pharmacy, Kyung Hee University, Seoul 02447, Korea; 7 Department of Molecular Science and Technology, Ajou University, Suwon 16499, Korea; ehkim01@ajou.ac.kr; 8 Department of HY-KIST Bio-Convergence, Hanyang University, Seoul 04763, Korea

**Keywords:** cancer immunotherapy, type I interferon, STING, STING activator

## Abstract

In cancer immunotherapy, the cyclic GMP–AMP synthase–stimulator of interferon genes (STING) pathway is an attractive target for switching the tumor immunophenotype from ‘cold’ to ‘hot’ through the activation of the type I interferon response. To develop a new chemical entity for STING activator to improve cyclic GMP-AMP (cGAMP)-induced innate immune response, we identified KAS-08 via the structural modification of DW2282, which was previously reported as an anti-cancer agent with an unknown mechanism. Further investigation revealed that direct STING binding or the enhanced phosphorylation of STING and downstream effectors were responsible for DW2282-or KAS-08-mediated STING activity. Furthermore, KAS-08 was validated as an effective STING pathway activator in vitro and in vivo. The synergistic effect of cGAMP-mediated immunity and efficient anti-cancer effects successfully demonstrated the therapeutic potential of KAS-08 for combination therapy in cancer treatment.

## 1. Introduction

In the current state of cancer therapy, immune checkpoint inhibitors (ICIs), such as anti-PD-1, anti-PD-L1, and anti-CTLA4, have emerged as one of the most successful strategies in clinics and have used in the market to cure cancer [1]. ICIs block tumor cells escaping from immune surveillance and induce tumor-cell death by increasing the recognition of tumor antigens and the generation of tumor-specific cytotoxic T lymphocytes (CTLs) [2]. Thus, intrinsic anti-tumor immunity in patients is considered a key factor in the success of ICIs. Despite the optimistic results for the ICIs, the overall response rate remains insufficient [3]. In the case of advanced melanoma, less than 10% of patients have shown a complete response rate [4]. The lack of CTLs and anti-tumor immunity in the tumor microenvironment is considered to be one of the main reasons for the low response rate of ICIs [5]. To address this, recent cancer immunotherapy has focused on the stimulation of the innate immunity to encourage a CTL-rich tumor microenvironment, resulting in T cell-inflamed tumor immunophenotype [6,7]. From this point of view, the activation of the cyclic GMP–AMP synthase (cGAS)–stimulator of interferon genes (STING) pathway to induce an inherent type I interferon (IFN) response has received increasing attention as the next generation of cancer immunotherapy [8,9,10]. Based on the critical role of the type I IFN response in stimulating T cell cross-priming [2,11,12,13], the activation of the STING pathway has been highlighted as a promising target to change immunologically ‘cold’ tumors to ‘hot’ tumors, thereby rendering tumors more susceptible to checkpoint blockades [9,13,14].

STING is known as a cytoplasmic DNA sensor that detects the second messenger, cyclic GMP-AMP (cGAMP), produced by cGAS in response to foreign DNA from bacteria, DNA viruses, and self-DNA, from leaking mitochondria or dead cells without any sequence specificity [15]. STING is located in the endoplasmic reticulum (ER) membrane in the basal state [16]. Once cGAMP binds to STING, it is activated and translocated to the ER-Golgi intermediate compartment (ERGIC), Golgi, or perinuclear area. Thereafter, the downstream signaling cascade is initiated by the phosphorylation of TANK-binding kinase 1 (TBK1) [15,17,18]. Phosphor-TBK1 induces the phosphorylation of STING, followed by the recruitment and phosphorylation of interferon regulatory factor 3 (IRF3) [19]. Then, phospho-IRF3 forms a homodimer and moves into the nucleus to trigger IFNB gene transcription, eventually activating type I IFN response by promoting various IFN-stimulated gene (ISG) expression [20,21].

In an effort to pharmacologically activate the STING pathway, studies on natural cyclic dinucleotide (CDN) ligands (e.g., c-di-GMP, c-di-AMP, 3,3-cGAMP, and 2,3-cGAMP) have led to the development of a synthetic CDN, ADU-S100 (dithio-(Rp, Rp)-[cyclic[A(2′,5′)pA(3′,5′)p]]) revealed the high potency of STING stimulation [22,23]. Based on its remarkable activity in immunity and anti-tumor efficacy in preclinical studies, ADU-S100 was successfully launched in a phase II clinic in combination with Pembrolizumab [24,25]. However, the clinical trial of ADU-S100 was terminated with unsatisfactory efficacy in 2019 [26]. Presumably, troubleshooting in dosage resulted from a bell-shaped response, a restricted administration route by intratumor injection, and a side effect of provoking inflammation in normal tissue due to ubiquitous STING expression, which may limit its applications and clinical feasibility [27,28]. To overcome this, there is a growing effort to develop new small-molecule STING activators, STING agonists, or STING pathway activators [29,30]. However, the lack of new chemical entities has impeded STING-activating drug discovery.

In this study, we demonstrate the new role of an old drug candidate, DW2282, as a STING activator. Structural modification allowed us to develop a novel STING activator, KAS-08, to boost the cGAMP-induced type I IFN response and STING signaling cascade in both human and mouse cells. Mechanistic studies clarified the differentiated mode of action of DW2282 and KAS-08 for STING activation. Further in vivo analysis suggests the therapeutic potential of KAS-08 as a synergistic drug for the combination of cancer immunotherapy.

## 2. Materials and Methods

### 2.1. Chemistry

The general synthetic procedures are described in the Supplementary Materials. A detailed synthetic method, yield, characterization, and NMR spectra of the intermediates and the final compounds are also reported in the Supplementary Materials.

### 2.2. Cell Culture and Reagents

The human monocyte cell lines THP1-Dual™, THP1-Dual™ KO-STING, and THP1-Dual™ KO-TBK1 were purchased from InvivoGen and cultured in RPMI1640 with 2.05 mM L-glutamine (Hyclone, Logan, UT, USA) supplemented with 10% heat-inactivated fetal bovine serum (FBS) (Hyclone), 1% penicillin/streptomycin (Corning, New York, NY, USA), and 0.2% Normocin (InvivoGen, San Diego, CA, USA). Mouse macrophage Raw264.7 cells and mouse colorectal cancer CT26 cells were purchased from KCLB and cultured in DMEM supplemented with 10% heat-inactivated FBS, 1% penicillin/streptomycin, L-glucose, L-glutamine and sodium pyruvate. All of the cell lines were maintained at 37 °C with 5% CO_2_ in a humidified incubator.

### 2.3. ISG Reporter Assay

The THP-1 cells were incubated in 384-well plates with the indicated concentrations of compounds for 30 min. Then, cGAMP (1 μg/mL) was added to the assay medium for a further 24 h. The supernatant was treated with the luminescence reagent QUANTI-Luc^TM^ (rep-qlc2; InvivoGen), according to the manufacturer’s protocol. The luminescence signal was measured using a microplate reader (SPARK; Tecan, Männedorf, Switzerland). The results were analyzed using GraphPad Prism 9 (Version 9.0.0, GraphPad Software, Inc., San Diego, CA, USA) and TIBCO Spotfire Analyst (Version 10.10.1, TIBCO Spotfire, Palo Alto, CA, USA).

### 2.4. High-Throughput Screening (HTS)

The chemical library for HTS and preliminary structure-activity relationship (SAR) screening were kindly provided by the Korea Chemical Bank. Individual compounds were treated in a 384-well plate using 96 Multi-Blot floating pin replicator. Briefly, 0.2 µL of all the compounds were added to 50 µL of growth medium containing THP-1 cells. After 30 min, cGAMP (1 µg/mL) was added to the assay medium for a further 24 h. The supernatant was transferred to a new 384-well plate using S3 Pipettor 384 (Apricot Designs, Covina, GA, USA). The screening read-out was measured using the ISG reporter assay according to the above-mentioned ISG reporter assay protocol.

### 2.5. ELISA

Cells were incubated in 96-well plates with the indicated concentrations of compounds prior to cGAMP (1 μg/mL) stimulation. The conditioned media were collected after 24 h. Human or mouse IFN-β and IP-10 were quantified using the DuoSet ELISA kit (DY814-05 and DY266-05; R&D Systems, Minneapolis, MN, USA), according to the manufacturer’s protocol.

### 2.6. Western Blot Analysis

The THP-1 cells were harvested and lysed after treatment with compounds. The proteins were extracted using an RIPA buffer (BIOSESANG, Seongnam, Korea) containing protease and phosphatase inhibitor cocktail (Thermo Fisher, Waltham, MA, USA). Protein lysates were electrophoresed on 9% SDS-PAGE gels and transferred onto PVDF membranes. The membrane was blocked with 5% BSA in Tris-buffered saline with Tween-20 (TBST; BIOSESANG) solution for 1 h at room temperature, followed by washing with TBST. Membranes were incubated with the following antibodies: (1) at 1:1000 dilution—phosphorylated STAT1(Tyr701) (#7649; Cell Signaling, Danvers, MA, USA), phosphorylated TBK1 (Ser172) (#5483; Cell Signaling), phosphorylated IRF3 (Ser396) (#4947S; Cell Signaling), phosphorylated STING (S366) (#19781; Cell Signaling), STAT1 (#9172; Cell Signaling), IRF3 (#11904; Cell Signaling), TBK1 (#3504; Cell Signaling), STING (#13647; Cell Signaling), and (2) at 1:3000 dilution—β-actin (#4970; Cell Signaling). HRP-conjugated secondary antibody (#7074; Cell Signaling) was used at a dilution of 1:3000. Luminescent images were visualized using the ChemiDoc Imaging System (Bio-Rad, Hercules, CA, USA).

### 2.7. STING Binding Assay

For the competitive STING-binding assay, the recombinant STING protein and HTRF-based assay kit were used (64BDSTGPEG; cisbio, Codolet, France). Individual compounds or standards were dispensed into the wells of a white 384-well plate. Then, human STING WT protein 6His-tagged was added to the same wells, followed by the addition of premixed STING WT ligand d^2^ reagent and 6His-Tb antibody working solution. The plate was incubated for 3 h at room temperature, avoiding exposure to light, after which the HTRF signal was measured using an HTRF-compatible reader.

### 2.8. Cellular Thermal Shift Assay (CETSA)

THP-1 cells were incubated with the indicated concentrations of DW2282 or KAS-08 for 3 h. After harvesting, the cells were divided into equal volumes and heated at a specific temperature for 3 min. Proteins were extracted with an RIPA buffer (BIOSESANG) containing protease and phosphatase inhibitor cocktail (Thermo Fisher). Protein lysates were electrophoresed on 9% SDS-PAGE gels and transferred onto PVDF membranes. The membrane was blocked with 5% BSA in Tris-buffered saline with Tween-20 (TBST) solution for 1 h at room temperature, followed by washing with TBST. The membranes were analyzed according to the above-mentioned Western blot analysis protocol.

### 2.9. CT26 Mouse Tumor Model Test

Eight-week-old female BALB/c mice (DBL, Dae Han Bio Link Co., Eumseong-gun, Korea) were used for in vivo experiments. Mice were inoculated subcutaneously with 1 × 10^6^ CT26 tumor cells in PBS on the right flank. Following tumor implantation, mice were randomized into four different treatment groups when the tumor reached 50–100 mm^3^. On day 5 after tumor cell injection, KAS-08 or vehicle was administered by intravenous injection (15 mg/kg) or vehicle (5% DMSO, 5% Tween-80 in PBS). 2′3′-cGAMP (Invivogen) was injected by intratumoral injection (2 μg in PBS). Tumor and body weight measurements were performed using digital calipers and weighing scales, respectively. Tumor volume was estimated using the following formula: tumor volume = (length × width^2^)/2. Mice were euthanized when the tumor volume approached approximately 2000 mm^3^. Tumor samples were collected at the indicated time points.

### 2.10. Statistical Analysis

All cell-based experiments were performed in triplicate, and a statistical analysis was performed by a Student’s *t*-test between the control group and the high concentration treatment group (*n* = 3). For the in vivo experiments, a statistical analysis was performed using a paired *t*-test for statistics (*n* = 4–5). All statistical analyses were performed using the GraphPad Prism software.

## 3. Results

### 3.1. Identification of Hit Compounds from Phenotypic Screening for cGAMP-Mediated Immune Response

To identify a new chemical entity for STING activators, we performed an HTS targeting of cGAS-STING pathway activation using a luciferase assay. THP-1 human monocyte cells harboring an IRF–inducible luciferase reporter construct (ISG reporter) were used throughout the screening. Because we aimed to discover new small-molecule activators to elevate the STING-mediated immune response, phenotypic screening was conducted by monitoring ISG reporter signals in the presence of a marginal dose of cGAMP (1 μg/mL = 1.39 μM) using a random chemical library containing approximately 7000 compounds. Since 2,3-cGAMP is the only cGAMP produced in mammalian cells, we used 2,3-cGAMP as a STING ligand to provoke an immune response in HTS and further biological evaluation [31].

**Figure 1:**
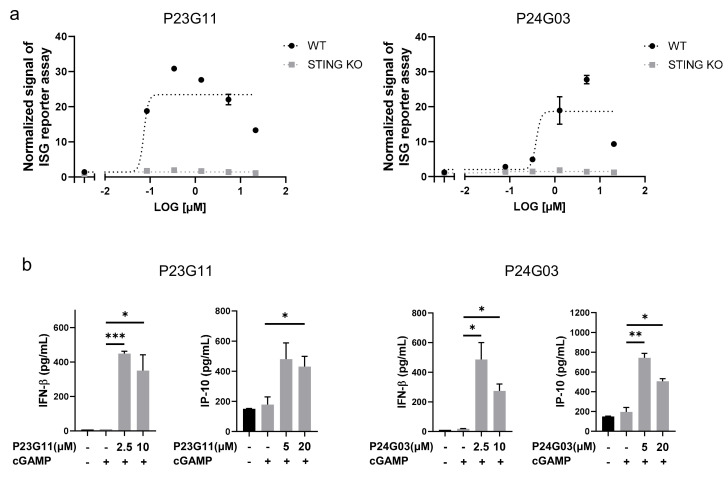
Identification of small-molecule STING activators via phenotypic screening. (**a**) ISG reporter assay of P23G11 and P24G03 in presence of cGAMP (1 μg/mL) in THP-1 WT and STING KO cells. Luciferase signal was normalized by DMSO control. (**b**) Measurement of IFN-β and IP-10 secretion by ELISA. Compounds were treated in THP-1 cells for 24 h using the indicated concentration. Graphs show the mean and standard deviation (SD). *: *p* < 0.05, **: *p* < 0.01, and ***: *p* < 0.001 by Student’s *t*-test.

The HTS campaign for boosting the cGAMP-induced immune response identified two interesting compounds, P23G11 and P24G03. From the dose–response validation, both P23G11 and P24G03 burst the ISG reporter signal in THP-1 WT cells, while no effect was observed in STING KO cells that confirmed STING-dependent activation of these two compounds (E_max_ for P23G11 = 23.7 ± 5.5 and E_max_ for P24G03 = 18.7 ± 7.5 in Figure 1a). To further confirm the innate immune response, we monitored the enhancement of cytokine release, such as IFNβ and IP-10, and both P23G11 and P24G03 clearly elevated the secretion of type I IFN cytokines by co-treatment with cGAMP (*p* = 0.0004 at 2.5 μM and *p* = 0.0343 at 10 μM in IFNβ; *p* = 0.0516 at 20 μM in IP-10 of P23G11; *p* = 0.0283 at 2.5 μM and *p* = 0.0162 at 10 μM in IFNβ; *p* = 0.0066 at 5 μM and *p* = 0.0139 at 20 μM in IP-10 of P24G03 in Figure 1b). These results suggested the potential use of two compounds as early hit compounds.

### 3.2. SAR Analysis Identified KAS-08 as a New STING Activator

**Figure 2:**
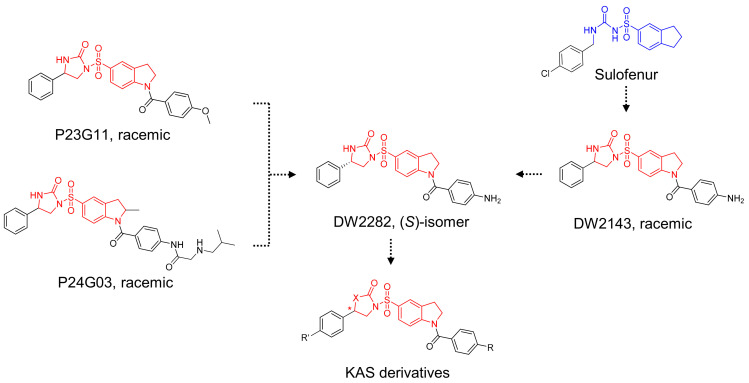
Schematic diagram of chemical structure for the design of KAS derivatives.

Based on the structural insights gained from P23G11 and P24G03, we hypothesized that diarylsulfonylurea would be a pharmacophore of the desired phenotype (Figure 2). For further SAR studies, we focused on the structure of DW2282, which was previously reported as an anti-tumor agent modified from Sulofenur and DW2143, but which was dropped during the preclinical stage due to gastrointestinal toxicity (Figure 2) [32,33,34,35,36,37].

A comprehensive review of the previous reports allowed us to evaluate DW2282 and its derivatives for the synergistic effect in the presence of low-dose cGAMP. For the preliminary screening, we explored the SAR of the four compounds using ISG reporter assay in WT and STING KO cells (Tables S1 and S2, Figures S1 and S2). All of the active compounds revealed STING-dependent activity and no stimulation of cGAMP-induced immune response in STING KO cells (Table S1 and Figure S1). Keeping the imidazolidinone core, variations of substituents in the acyl indoline moiety suggested that the aryl group played an essential role in stimulating the immune response in the presence of cGAMP (R^1^ in Table S1, entries 1–12 vs. 13–17). We found that the *para*-amino phenyl-substituted compound KAS-S07 (DW2143) and the 3-pyridiyl compound KAS-S10 showed promising effects on activating STING (Table S1, entries 7 and 10). Substituents on the phenyl group at the imidazolidinone were also critical, as exemplified by the chloro-substituted compound (R^2^ in Table S1, entries 5 and 15 vs. 6 and 16). In addition, pyrrolidinone-based compounds completely lost their desired effect, emphasizing the importance of the cyclic urea-based imidazolidinone scaffold (Table S2 and Figure S2).

Based on the STING activating efficacies of KAS-S07 (DW2143) and KAS-S10, we established a focused library of ten compounds and examined their effects on boosting the cGAMP-induced immune response (Table 1 and Figure S3). Based on previous reports comparing DW2143 and DW2282, we decided to prepare each (*R*)- and (*S*)-stereoisomers for all the designed compounds and compare their stereo-specific effects.

The SAR results for the newly designed compounds are summarized in Table 1. Based on the preliminary screening results, we first evaluated the stereoisomer effect of the racemic mixture, KAS-S07 and KAS-S10. Consistent with a previous report on stereoisomers for DW2143 [36], only (*S*)-isomers, such as DW2282, KAS-02, KAS-04, KAS-06, and KAS-08, showed an increase in cGAMP-induced ISG reporter activity, while no effective signals were measured in their (*R*)-isomers, such as KAS-01, KAS-03, KAS-05, KAS-07, and KAS-09 (Table 1). A 4-pyridyl compound, KAS-02, maintained its activity but exhibited reduced potency compared to DW2282. Then, we synthesized aryl-substituted compounds with nitrogen-based small functional groups at the *para* position of the aryl ring. The replacement of the amino group with a hydroxylamine enhanced the EC_50_ value, but decreased the E_max_ value compared to the amino group (KAS-06 vs. DW2282). Interestingly, KAS-08, prepared by the introduction of a nitro group at the *para* position, showed retained STING-activating capability with the minimization of the perturbed potency (330 nM of EC_50_ and 41.4-fold of E_max_). Overall, DW2282 and KAS-08 provided potent and effective activities to amplify cGAMP-induced reporter signals, suggesting their potential applicability as synergistic drugs that promote the STING signaling pathway.

### 3.3. Investigating the Mode-of-Action of DW2282 and KAS-08 on STING-Mediated IFN Response

**Figure 3:**
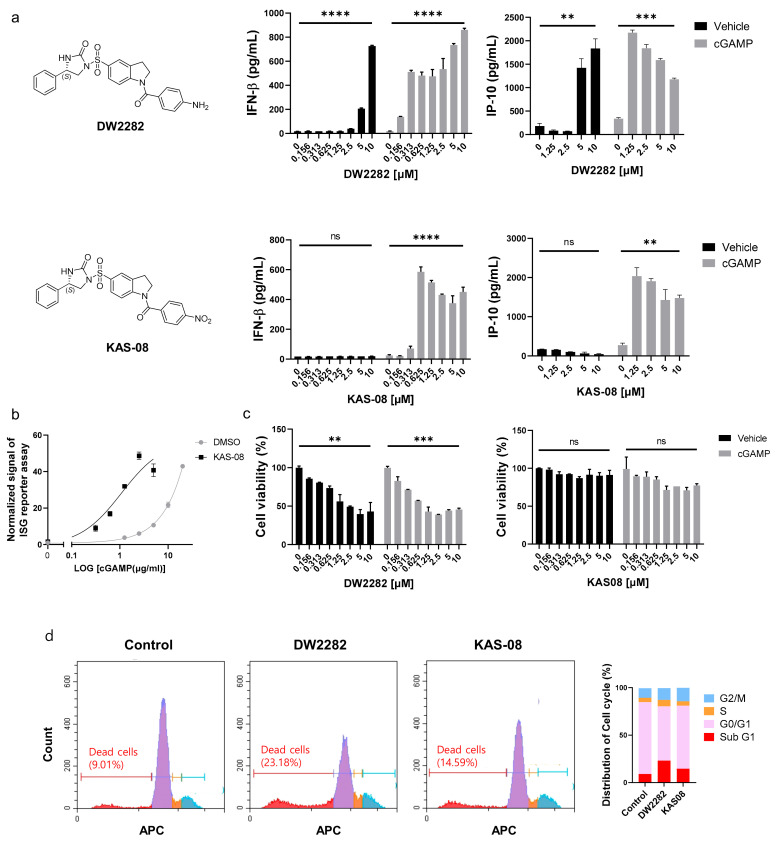
Differentiated mode-of-action between DW2282 and KAS-08 for boosting cGAMP-induced immunity. (**a**) Chemical structure of two compounds and ELISA analysis of IFNβ and IP-10 by DW2282 (**up**) and KAS-08 (**down**) treatment in the absence or presence of cGAMP (1 μg/mL) in THP-1 cells. (**b**) ISG reporter assay by titration of cGAMP in absence or presence of KAS-08 (2.5 μM) in THP-1 cells. (**c**) Cell viability after treatment of DW2282 (**left**) and KAS-08 (**right**) for 24 h in THP-1 cells. Graphs show the mean and SD. **: *p* < 0.01, ***: *p* < 0.001, ****: *p* < 0.0001, and ns: non-significant by Student’s *t*-test. (**d**) Cell cycle analysis measured by FACS. THP-1 cells were treated by DW2282 (10 μM) and KAS-08 (10 μM) for 6 h. Histogram graphs (**left**) and the quantified result (**right**).

After establishing the hit compounds, we analyzed IFNβ secretion in the absence or presence of cGAMP to validate the synergistic effect on the type I IFN response. Surprisingly, DW2282 and KAS-08 exhibited different modes of action in cytokine secretion. Consistent with the reporter assay (Table 1 and Figure S3), the co-treatment of each compound with cGAMP enhanced the marginal response of low-dose cGAMP and resulted in remarkable cytokine release for IFNβ and IP-10 (*p* = 0.0001 at 10 μM of DW2282 with cGAMP in IFNβ; *p* = 0.0008 at 10 μM of DW2282 with cGAMP in IP-10; *p* < 0.0001 at 10 μM of KAS-08 with cGAMP in IFNβ; *p* = 0.0026 at 10 μM of KAS-08 with cGAMP in IP-10 in Figure 3a). These results confirmed the robust synergistic effects of DW2282 and KAS-08. Notably, KAS-08 showed no effect on IFNβ and IP-10 secretion by a single treatment, whereas DW2282 induced IFNβ and IP-10 production by itself at high concentrations (*p* < 0.0001 at 10 μM DW2282 without cGAMP in IFNβ; *p* = 0.0083 at 10 μM DW2282 without cGAMP in IP-10 in Figure 3a). Due to the titration of cGAMP, KAS-08 clearly improved the signal window of cGAMP (EC_50_ of cGAMP = 31.25 μg/mL and EC_50_ cGAMP KAS-08 = 3.799 μg/mL in Figure 3b). With these results, we confirmed that KAS-08 amplified the activity of cGAMP, but no stimulation on type I IFN response by itself.

Since cellular toxicity and DNA leakage by DW2282 have been reported previously [37,38], we postulated that self-DNA leakage from micronucleus and mitochondria from DNA-damaged cells allowed for the activation of cGAS to produce cGAMP, which provoked DW2282-induced STING activation without additional cGAMP treatment [39]. To validate this hypothesis, cell viability was evaluated in THP-1 cells by DW2282 and KAS-08 treatment in the presence or absence of cGAMP. Additionally, DW2282 clearly reduced cell viability regardless of cGAMP stimulation, but KAS-08 showed no critical cellular toxicity (*p* = 0.0052 and *p* = 0.0006 of DW2282 in the absence or presence of cGAMP in Figure 3c). We further investigated the cell cycle arrest to elucidate the intracellular DNA fragmentation induced by DW2282 and KAS-08. As a result, DW2282 induced cell cycle arrest in the G/M phase and substantially increased Sub-G1 phased cells within 6 h, supporting the loss of nuclear DNA content by DNA fragmentation (Figure 3d). These data suggest that DW2282 facilitated the stimulation of STING by itself using cGAMP produced by self-DNA, resulting in cell cycle arrest. Moreover, DW2282 and KAS-08 both contributed to STING-pathway activation to generate a synergistic effect with cGAMP.

### 3.4. Direct STING-Binding Property of DW2282

**Figure 4:**
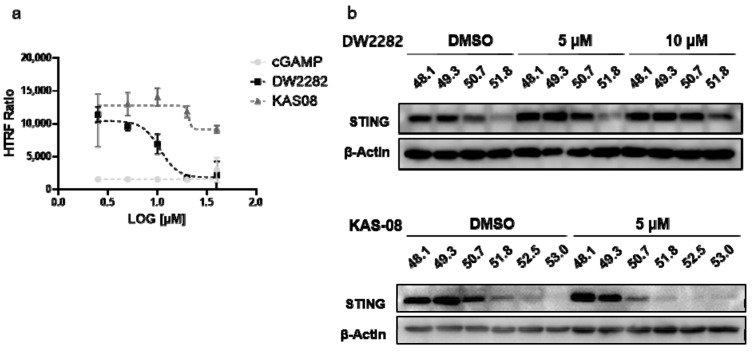
DW2282 stimulated STING by direct binding. (**a**) Analysis of ligand-competitive binding assay in vitro between human recombinant STING protein and DW2282 or KAS-08. The homogeneous time resolved fluorescence (HTRF) ratio indicates the interaction between STING protein and cGAMP-modified ligand. (**b**) Cellular thermal shift assay of DW2282 and KAS-08 for STING protein. DW2282 and KAS-08 were treated in THP-1 cells with cGAMP (1 μg/mL) for 3 h, then subjected to heating by indicated temperature.

Although DNA fragmentation induced by DW2282 was found to be responsible for provoking STING activation, we wanted to clarify whether the STING protein is a direct target of DW2282. To this end, we performed an in vitro ligand-competitive binding assay using a human STING recombinant protein and STING ligand-d^2^. Interestingly, DW2282 competed with the STING ligand at a high concentration (>10 μM), indicating that DW2282 directly binds with STING at cGAMP-binding site, while KAS08 showed no critical effect (15,119.0 ± 0.3 in DMSO control, 1516.3 ± 7.1 at 20 μM of cGAMP, 1813.3 ± 13.4 at 20 μM of DW2282, and 11,847.2 ± 7.3 at 20 μM of KAS-08 in Figure 4a). For further verification, a cell-based thermal shift assay was performed in THP-1 cells to evaluate the thermal stability through the interaction between the small molecule and target protein. Consistent with the in vitro binding results, DW2282 increased the stabilization of STING protein in cells with increasing temperature (Figure 4b). Additionally, KAS-08 was not able to induce any benefit for protein stabilization (Figure 4b), which implied that KAS-08 only stimulated the STING pathway and not directly bound to STING. Overall, we confirmed that DW2282 binds STING directly at high concentration, not KAS-08.

### 3.5. Elucidation of STING Signaling Pathway Regulated by DW2282 and KAS-08

**Figure 5:**
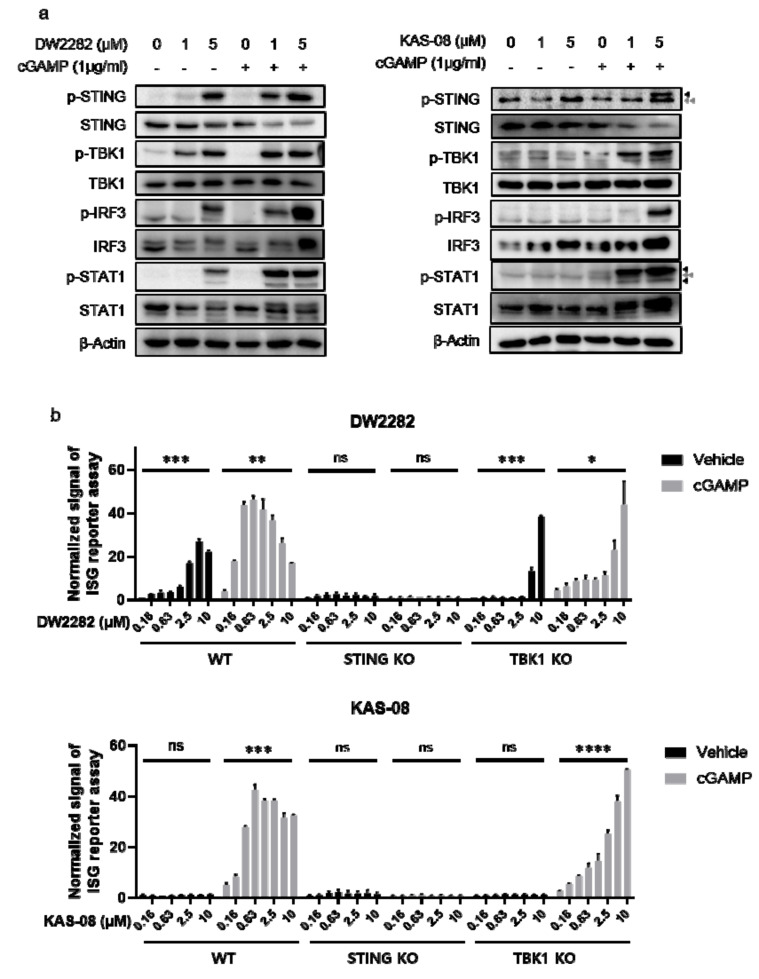
Analysis of the signaling pathway for cGAMP-mediated STING activation by DW2282 and KAS-08. (**a**) Western blot analysis for STING signaling pathway. Both DW2282 and KAS-08 were treated in THP-1 cells for 6 h in the absence or presence of cGAMP (1 μg/mL) stimulation. Black arrowheads indicate p-STING or p-STAT1 and gray double-arrowheads indicate non-specific band. (**b**) ISG reporter assay of DW2282 (**up**) and KAS-08 (**down**) in THP-1 WT, STING KO, and TBK1 KO cells. DW2282 and KAS-08 were treated in the range from 10 μM to 160 nM in the absence or presence of cGAMP (1 μg/mL). Luciferase signal was normalized by DMSO control. Graphs show the mean and SD. *: *p* < 0.05, **: *p* < 0.01, ***: *p* < 0.001, ****: *p* < 0.0001 and ns: non-significant by Student’s *t*-test.

To investigate the mechanism of action for both compounds, we monitored the activation of downstream signaling proteins. As predicted from the results of cytokine secretion (Figure 3a), DW2282 and KAS-08 were distinguished in the mechanism of STING pathway activation. We found that DW2282 initiated the STING signaling pathway by itself, resulting in the phosphorylation of STING, TBK1, IRF3, and STAT1 as biomarkers for STING and IFNR signaling, whereas a single treatment of KAS-08 hardly increased phosphorylation of the associated pathway compared to control or low dose of cGAMP (Figure 5a). Upon co-treatment with 1 μg/mL of cGAMP, which was too low to initiate the STING signaling cascade, DW2282 and KAS-08 assisted in the phosphorylation of STING and downstream effectors, TBK1 and IRF3, by amplifying the signal window of cGAMP (Figure 5a). The enhanced therapeutic window of DW2282 and KAS-08 contributed not only to the STING-TBK1-IRF3 cascade but also to IFNR-related type I IFN signaling confirmed by phosphor-STAT1 (Figure 5a). We further validated that the DW2282 and KAS-08-induced synergy effect was not related to preventing STING from its degradation, since the level of STING was decreased by co-treatment with cGAMP and both compounds (Figure 5a). These results suggest that the synergistic effect induced by DW2282 and KAS-08 is related to the regulation of phosphorylation of the STING-TBK1 complex.

For further clarification, a loss-of-function study using THP-1 STING KO and TBK1 KO cells was performed using the ISG reporter assay. Interestingly, the fact that the increase in ISG signal by DW2282 and KAS-08 with cGAMP co-treatment was completely STING-dependent but partially dependent on TBK1 suggests that IκB-NFκB signaling is involved in the regulatory mechanism of DW2282 and KAS-08 synergy as a compensatory mechanism (*p* = 0.0354 at 10 μM of DW2282 with cGAMP in TBK1 KO cells and *p* < 0.0001 at 10 μM of KAS-08 with cGAMP in TBK1 KO cells in Figure 5b) [40,41]. These results indicate that the amplification of STING signaling by DW2282 and KAS-08 was responsible for the upregulation of phosphor-STING acting upstream of TBK1. Furthermore, in the case of DW2282, both the direct binding to STING and the increase in the phosphor-STING level were the underlying causes of DW2282-mediated STING activation.

### 3.6. KAS-08 Activated cGAMP-Mediated Immunity for Mouse STING

The main reason that DMXAA failed in clinical trials was that DMXAA only activates mouse STING, not human STING [42,43]. For this reason, the broad reactivity in mammals is considered to be an important issue in the discovery of small molecule-based STING activators.

**Figure 6:**
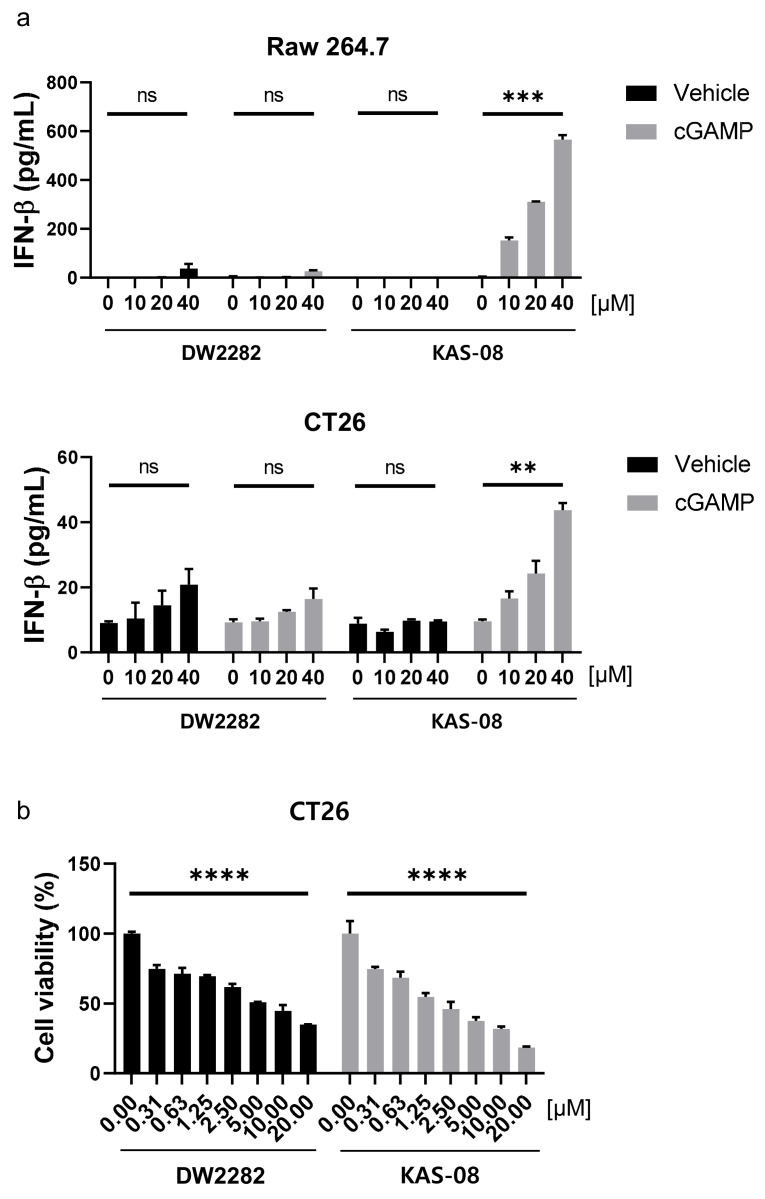
KAS-08 enhanced the cGAMP-mediated STING activation in mouse cells. (**a**) ELISA analysis of IFNβ secretion by DW2282 and KAS-08 treatment in the absence or presence of cGAMP (1 μg/mL) in Raw264.7 (**up**) and CT26 (**down**) cells. (**b**) Cell viability of DW2282 and KAS-08 after 72 h treatment in CT26 cells. Graphs show mean and SD. **: *p* < 0.01, ***: *p* < 0.001, ****: *p* < 0.0001 and ns: non-significant by Student’s *t*-test.

To evaluate the potential availability of DW2282 and KAS-08 in vivo, we investigated the activity of both compounds in mouse cells. In Raw264.7 murine macrophage cells and CT26 murine colorectal carcinoma cells, KAS-08 clearly boosted cGAMP activity in both mouse cells (*p* = 0.0005 at 10 μM of KAS-08 with cGAMP in Raw264.7 cells and *p* = 0.0024 at 10 μM of KAS-08 with cGAMP in CT26 cells) in Figure 6a). Although DW2282 markedly induced STING activation in human THP-1 cells (Figure 3), it had a negligible effect on IFNβ secretion in mouse cells (Figure 6a). These results revealed the reason for the low in vivo efficacy of DW2282 in a previous report. Only 20% of tumor growth suppression was observed in CT26-inoculated mice by DW2282 treatment, presumably due to insufficient immune activation in mice [36]. On the other hand, a further evaluation of cell viability against CT26 cancer cells confirmed that KAS-08 exhibited cellular toxicity (*p* < 0.0001 at 10 μM of DW2282 and *p* < 0.0001 at 10 μM KAS-08 in Figure 6b). We assumed that cGAMP released from cancer cells initiated STING activation, which contributed to the effect of KAS-08 [44]. Based on the overall results of DW2282, including the safety issues related to immune cell toxicity and gastrointestinal toxicity, the low activity on mouse cells, and the marginal in vivo efficacy from the CT26 colorectal tumor model (Figure 3a and Figure 6a) [36,37], KAS-08 was chosen for further in vivo anti-cancer activity study.

### 3.7. Anti-Cancer Efficacy of KAS-08 in CT26-Bearing Syngeneic Tumor Model

**Figure 7:**
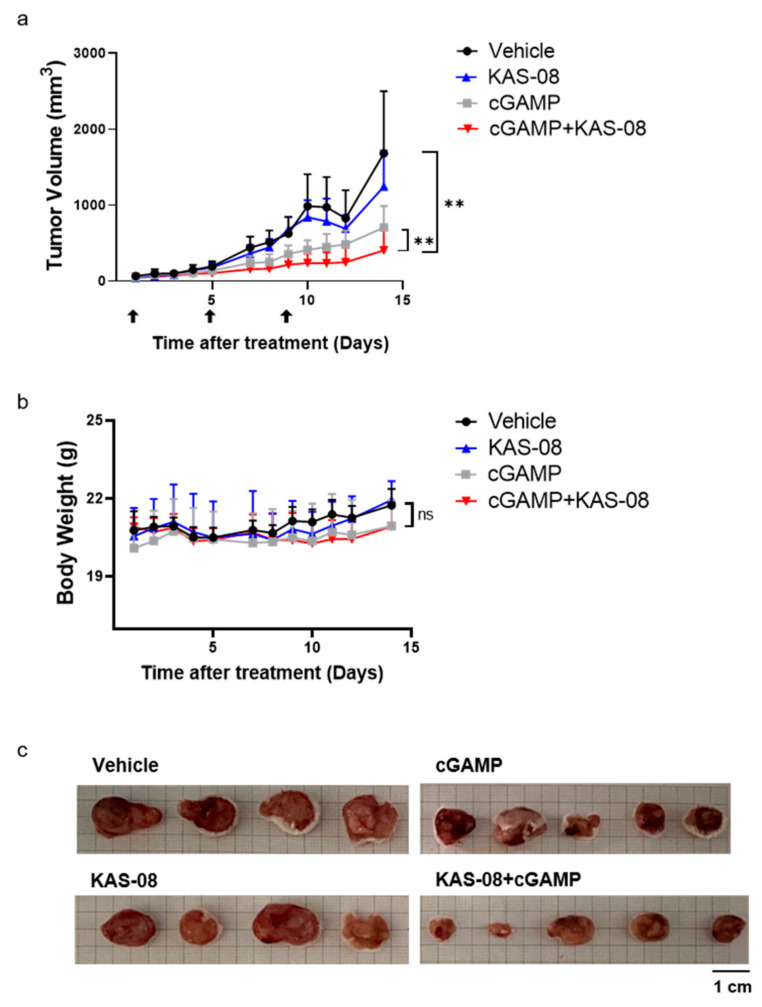
Evaluation of the synergy anti-cancer effect of KAS-08 by combination with cGAMP in CT26 bearing mouse. Balb/c mice were administrated by four different groups. Vehicle (*n* = 4), KAS-08 (*n* = 4, 15 mg/kg, i.v.), cGAMP (*n* = 5, 2 μg, i.t.), and KAS-08 (*n* = 5, 15 mg/kg, i.v.) with cGAMP (2 μg, i.t.). cGAMP was injected three times at the indicated days. KAS-08 was injected eight times every one or two days. (**a**) Tumor volume of four different groups. Paired *t*-test for statistics. **: *p* < 0.01. (**b**) Change in body weight in the four different groups. Two-way ANOVA for statistics. ns: not significant. Graphs show the mean and SD. (**c**) Image of isolated tumor from mice after 14 days of monitoring.

To validate the in vivo anti-cancer efficacy, KAS-08 was studied in a CT26-bearing mouse syngeneic tumor model. CT26 in BALB/c is one of the most widely used in tumor models, not only for a syngeneic model, but also as a testing immunotherapeutic concept as a ‘cold’ tumor due to its modest immunogenic characteristics [40,41]. cGAMP is only available by intratumoral injection, which limits the broad application of therapy [45]. We expected that the newly developed KAS-08 overcame this challenging issue by stimulating innate immunity via endogenous cGAMP. However, the single administration of KAS-08 by intravenous injection showed no remarkable tumor suppression (Figure 7a). Considering the cGAMP titration result (Figure 3b), the lack of cGAMP release in CT26 cells was insufficient to initiate and amplify KAS-08-induced immunity. However, co-treatment of KAS-08 with cGAMP significantly suppressed tumor growth compared to vehicle or cGAMP only treatment (*p* = 0.0047 between vehicle and KAS-08 + cGAMP and *p* = 0.0027 between cGAMP only and KAS-08 + cGAMP in Figure 7a) without any significant body weight loss (Figure 7b). After 14 days of treatment, mice were sacrificed, and the tumor size was monitored to verify the anti-cancer effect of KAS-08 and cGAMP combination (Figure 7c). All these results supported the efficient synergy activity of KAS-08 as a combination agent with cGAMP in anti-cancer therapy.

## 4. Discussion

Since the cGAS-STING pathway plays a pivotal role in cancer immunotherapy by stimulating inherent type I IFN immunity, we aimed to discover a new chemical entity for STING activators. As a result, we successfully developed a STING pathway activator, KAS-08, as a synergistic drug candidate in combination with cancer therapy.

A sulfonyl urea scaffold was identified from the initial HTS. Based on the structural insights gained, we hypothesized that diarylsulfonylurea would be a pharmacophore of the desired phenotype. Sulofenur (LY186641) embedded with a diarylsulfonylurea skeleton was reported as an orally available anti-cancer and anti-neoplastic agent in the 1990s (Figure 2) [46]. Although sulofenur progressed to clinical trials, it was discontinued in a phase II clinical study due to its marginal efficacy and side effects such as methemoglobinemia and hemolytic anemia [32,33]. The ring construction of the urea affords 2-imidazolidinone, and the introduction of the *N*-acyl moiety in the sulfonyl indane of sulofenur led to the development of DW2143 (Figure 2). As a racemic mixture, DW2143 inhibited tumor growth both in vitro and in vivo [34,35]. Further identification of the *S*-enantiomer from DW2143 provided enantiopure DW2282, which improved tumor suppression compared to racemate in SW620 cells and SW620-xenograft nude mice [36]. However, DW2282 was not effective in CT26-bearing syngeneic mice with only marginal tumor suppression [36]. Despite its unknown mechanism of action, DW2282 has been studied as a potential candidate for use as an anti-tumor agent with higher specificity and lower toxicity than other orally active sulfonylureas [37]. Nonetheless, DW2282 was dropped in the preclinical assay due to gastrointestinal toxicity [36]. Our preliminary screening and further structural modification of DW2143 led to the development of DW2282 and KAS-08 as STING activators (Figure 2).

Despite their high structural similarity, DW2282 and KAS-08 activated STING with very different modes of action. We validated multiple roles of DW2282 in STING activation by: (1) the production of cGAMP induced by self-DNA from dead cells, (2) direct binding with STING, and (3) the amplification of the signal for a marginal dose of cGAMP, while KAS-08 only played the last role in enhancing STING activity initiated by cGAMP (Figure 3, Figure 4 and Figure 5). Although DW2282 provided the unique biological features of both the STING agonist and activator, the fact that DW2282-induced general toxicity on a broad range of cell panels with 0.095 µg/mL of mean GI_50_ (205 nM), as well as monocyte cells (Figure 3c), and the low activity in mouse cells (Figure 6a) and in vivo efficacy of DW2282, led us to focus on KAS-08 for further biological evaluation [36].

We confirmed that the KAS-08-mediated synergistic effect on cGAMP was presumably related to the upregulation of phospho-STING. Additionally, KAS-08 only negligibly activated and phosphorylated STING by itself without cGAMP, but was able to boost the type I IFN pathway once STING was activated (Figure 3, Figure 5 and Figure 6). In the downstream signaling pathway, STING-TBK1-IRF3 is considered to be a major signaling cascade for triggering IFNB transcription. However, STING-IKK-NFκB is also involved in the STING downstream pathway [47]. We found that KAS-08 improved cGAMP activity in TBK1 KO cells, even with less potency, but no activity in STING KO cells (Figure 5b). This moderate synergy effect in TBK1 KO cells suggests that the mechanism of KAS-08 is highly related to the upstream of TBK1 (Figure 5a). These results support that the upregulation of phosphor-STING by KAS-08 is responsible for the synergistic effect with cGAMP. To confirm this hypothesis, additional studies are needed to elucidate the molecular targets of KAS-08. For example, affinity-based target identification using chemical probe and MS analysis or large-scale genetic screening [48,49]. If the target of KAS-08 is validated, this will provide a new therapeutic target for cancer immunotherapy.

In contrast to DW2282, KAS-08 showed no significant body-weight loss during the treatment period in vivo system (Figure 7b). Although we did not confirm all the side-effects against KAS-08, the low toxicity of KAS-08 in some type of immune cells such as THP-1 and PBMC (Figure 3c and Figure S4) and the minimal immune response without STING activation (Figure 3a) could provide an advantage in terms of a reduction in the potential side effects by targeting STING pathways in the tumor microenvironment.

Furthermore, our in vivo study demonstrated the anti-cancer efficacy of KAS-08 in combination with cGAMP using the CT26 tumor (Figure 7). Even though the single treatment of KAS-08 was not effective and the combination with cGAMP still required an intratumoral injection of cGAMP in the current study, we believe that the intravenous administration of KAS-08 extends the practical potential for general tumors in a future application. By investigating the cGAMP level in response to KAS-08 treatment (Figure 3b), KAS-08 could induce the desired effect if the cGAMP level in the tumor microenvironment is within the range that affecting KAS-08. Recent studies on the inhibition of ENPP or TREX have demonstrated the therapeutic potential of cGAS-STING pathway activators in combination with radiation therapy to increase cGAMP in tumors [44,50,51]. Likewise, further combinations, such as radiation therapy or other chemotherapies that promote cGAMP production, could provide new opportunities for the broad application of KAS-08. We hope that the STING activator identified in this study will provide new possibilities to overcome existing drawbacks, such as ICI-low response tumor or tumor resistance.

## 5. Conclusions

In conclusion, the phenotypic screening campaign identified diarylsulfonylurea analogues as STING pathway activators. Based on the SAR study and further biological evaluation, we elucidated the mechanism of an old drug candidate, DW2282, and presented a new chemical entity, KAS-08, for use as a combination reagent for cancer immunotherapy.

## Figures and Tables

**Table 1:** Structure-activity relationship study for DW2282 derivatives.

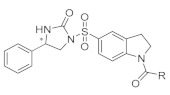				
Compound	Isomer	R	EC_50_ (μM) ^1^	E_max_^1^
DW2282	*S*	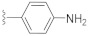	0.18	54.9
KAS-01	*R*	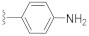	>10	N/A
KAS-02	*S*	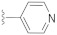	1.25	36.3
KAS-03	*R*	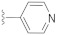	>10	N/A
KAS-04	*S*	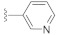	0.65	42.6
KAS-05	*R*	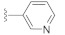	>10	N/A
KAS-06	*S*	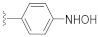	0.14	15.3
KAS-07	*R*	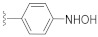	>10	N/A
KAS-08	*S*	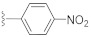	0.33	41.4
KAS-09	*R*	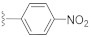	>10	N/A

1 Activity was measured using the ISG luciferase reporter assay in THP-1 cells. The individual compound was pre-treated before cGAMP (1 μg/mL) and then stimulated for 24 h. The luciferase signal was normalized using DMSO treatment. The EC_50_ and E_max_ were calculated using logistic regression. EC_50_ > 10 μM means no significant result within the tested dose range (10–0.04 μM). N/A, not applicable.

## Supplementary Materials

The following are available online at https://www.mdpi.com/article/10.3390/biomedicines10010033/s1, Table S1: Structure-activity relationships of 1-(indolin-5-ylsulfonyl)-4-phenylimidazolidin-2-ones; Table S2: Structure-activity relationships of 1-(indolin-5-ylsulfonyl)-4-phenylpyrrolidin-2-ones, Figure S1: Dose–response graph for Table S1, Figure S2: Dose–response graph for Table S2, Figure S3: Results for ISG reporter assay in Table 1. Figure S4: Result for cell viability of KAS-08 in PBMC. Chemical synthesis procedure, characterization, and NMR spectra.
