# Supplementary material for: Development of Small-Molecule STING Activators for Cancer Immunotherapy
Source: Biomedicines. 2021 Dec 24;10(1):33. doi: 10.3390/biomedicines10010033 (PMC8773171; doi:10.3390/biomedicines10010033)

Supplementary Materials for

Development of small-molecule STING activators for cancer immunotherapy

Hee Ra Jung [1,2,‡], Seongman Jo [3,4,‡] Min Jae Jeon [3,5], Hyelim Lee [1,6], Yeonjeong Chu [1,7], Jeehee Lee [1,8], Eunha Kim [7], Gyu Yong Song [4], Cheulhee Jung [2], Hyejin Kim [3,*] and Sanghee Lee [1,8,*]

[1] Creative Research Center for Brain Science, Brain Science Institute, Korea Institute of Science and Technology, Seoul 02792, Republic of Korea; 120044@kist.re.kr (H.R.J.); 120542@kist.re.kr (H.L.); tany828@kist.re.kr (J.L.)
[2] Department of Biotechnology, College of Life Sciences and Biotechnology, Korea University, Seoul 02841, Republic of Korea; damo363@korea.ac.kr (C.J.)
[3] Therapeutics & Biotechnology Division, Korea Research Institute of Chemical Technology, Daejeon 34114, Republic of Korea;
[4] Department of Pharmacy, College of Pharmacy, Chungnam National University, Daejeon 34134, Republic of Korea; jsm558@krict.re.kr (S.J.); gysong@cnu.ac.kr (G.Y.S.)
[5] Department of Medicinal Chemistry and Pharmacology, University of Science & Technology, Daejeon 34113, Republic of Korea; bluej936@krict.re.kr (M.J.J.)
[6] Department of Pharmacy, College of Pharmacy, Kyung Hee University, Seoul 02447, Republic of Korea
[7] Department of Molecular Science and Technology, Ajou University, Suwon, 16499, Republic of Korea; duswjd1415@ajou.ac.kr (Y.C); ehkim01@ajou.ac.kr (E.K.)
[8] Department of HY-KIST Bio-convergence, Hanyang University, Seoul 04763, Republic of Korea

‡ These authors equally contributed to this work.
* Correspondence: S.L. slee19@kist.re.kr; H.K. hjinkim@krict.re.kr

TABLE OF CONTENTS **Page**

1. Chemical Synthetic Procedures……….. 2-16

2. Preliminary screening results 17-20

3. Dose-response graphs for Table 1...21

4. Cell viability test for KAS-08..22

5. ^{1}H NMR spectrums……..23-27

1. Chemical Synthetic Procedures

1.1. General procedures

Unless otherwise stated, all reagents were purchased from Sigma-Aldrich, TCI, BLD pharm and used without further purification. *N,N*-dimethylformamide (DMF), CH_3CN, CH_2Cl_2, and tetrahydrofuran (THF) were dried and purified by using a JC Meyer solvent purification system before use. Analytical thin-layer chromatography (TLC) was used on Merck silica gel $60F^{254}$ glass plates. Purification by column chromatography was performed on silica-cartridges (Biotage Sfär Silica 60 μm) with a MPLC system. Compounds difficult to be isolated were purified using preparative TLCs. ^1H and ^{13}C NMR spectra were recorded on Bruker instruments (300 or 400 MHz for ^1H NMR, 101 MHz for ^{13}C NMR). Chemical shifts were quoted in parts per million (ppm) referenced to the appropriate solvent peak or 0.0 ppm for tetramethylsilane. The following abbreviations were used to describe peak splitting patterns when appropriate: s = singlet, d = doublet, t = triplet, q = quartet, dd = doublet of doublet, td = triplet of doublet, ddd = doublet of doublet of doublet, m = multiplet. Coupling constants, *J*, were reported in the hertz unit (Hz). High-resolution mass spectra (HRMS) were obtained by using Fast Atom Bombardment (FAB) ionization mode at 5 KeV, Resolution 5000. All compounds assayed were >95% pure, as determined by UPLC analysis conducted on Waters Acquity UPLC H-Class system with photodiode array (PDA) detector using a reverse-phase column with a linear CH_3CN/H_2O gradient system, 10% to 90% CH_3CN in H_2O.

1.2. Synthetic protocol and characterization for all reported compounds

(*R*)-1-(indolin-5-ylsulfonyl)-4-phenylimidazolidin-2-one, (core 1)

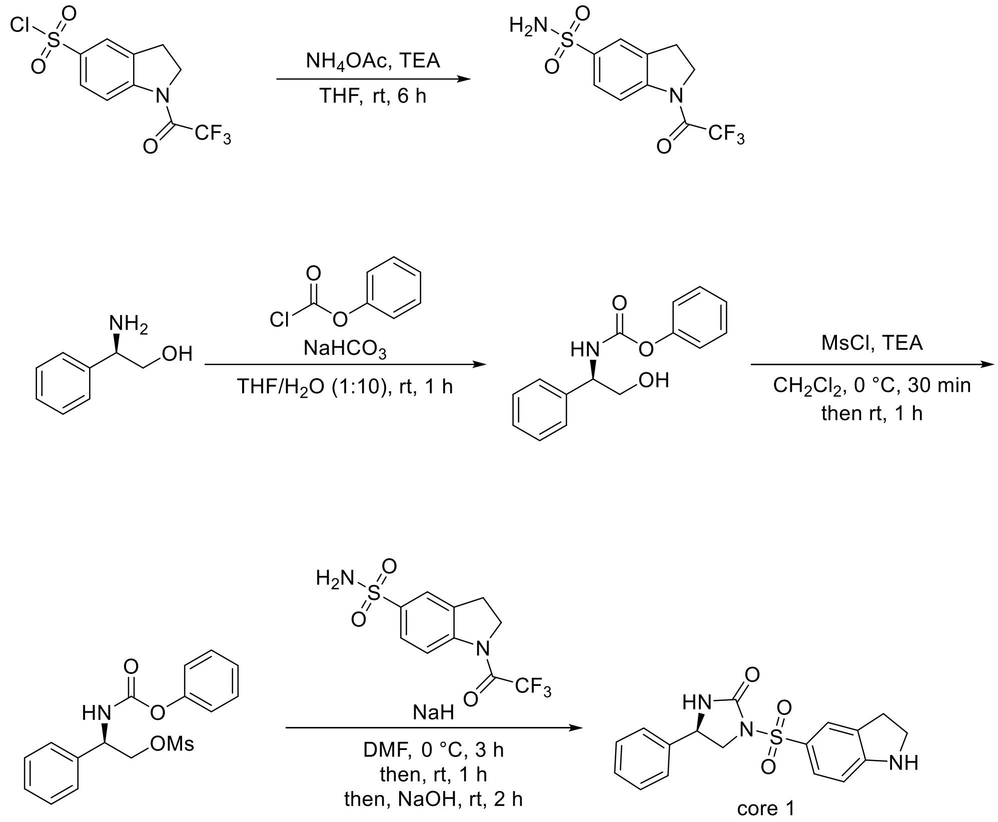

Step 1. Preparation of 1-(2,2,2-trifluoroacetyl)indoline-5-sulfonamide: To a solution of 1-(2,2,2-trifluoroacetyl)indoline-5-sulfonyl chloride (2.00 g, 6.38 mmol) in THF (128 mL, 0.05 M) were added NH_4OAc (6.88 g, 89.3 mmol) and trimethylamine (TEA) (13.3 mL, 95.7 mmol). After stirring the mixture for 6 h at room temperature, the reaction mixture was poured into water and extracted with EtOAc. The combined organic layer was washed with water and dried with $MgSO_4$ and concentrated *in vacuo*. The residue was purified by silica gel flash column chromatography (hexanes:EtOAc=1:1) to afford the title compound as a white solid (937 mg, 3.18 mmol, 51%). R_f 0.32 (hexanes:EtOAc=1:1).

^{1}H NMR (300 MHz, DMSO-d_6) δ 8.16 (d, *J* = 8.4 Hz, 1H), 7.79–7.71 (m, 2H), 7.37 (s, 2H), 4.34 (t, *J* = 8.3 Hz, 2H), 3.30 (d, *J* = 8.2 Hz, 2H).

Step 2. Preparation of phenyl (*R*)-(2-hydroxy-1-phenylethyl)carbamate: To a solution of (*R*)-(-)-2-phenylglycinol (1.00 g, 7.29 mmol) and sodium bicarbonate (0.919 g, 10.9 mmol) in H_2O (20 mL) was slowly added phenyl chloroformate (1.00 mL, 8.02 mmol) in THF (2 mL). After stirring the mixture for 1 h at room temperature, the mixture was poured into water and extracted with EtOAc. The combined organic layer was washed with water and dried with $MgSO_4$ and concentrated *in vacuo*. The residue was purified by silica gel flash column chromatography (CH_2Cl_2:MeOH=19:1) to afford the title compound as a white solid, which was used in the next step without further purification. R_f 0.32 (CH_2Cl_2:MeOH=19:1).

^{1}H NMR (400 MHz, DMSO-d_6) δ 8.25 (d, J = 8.6 Hz, 1H), 7.40–7.31 (m, 6H), 7.28–7.23 (m, 1H), 7.18 (t, J = 7.4 Hz, 1H), 7.08 (d, J = 7.9 Hz, 2H), 4.97 (t, J = 5.7 Hz, 1H), 4.63 (q, J = 7.3 Hz, 1H), 3.59 (t, J = 5.8 Hz, 2H).

Step 3. Preparation of (*R*)-2-((phenoxycarbonyl)amino)-2-phenylethyl methanesulfonate: To a solution of phenyl (*R*)-(2-hydroxy-1-phenylethyl)carbamate (1.50 g, 5.83 mmol) in CH_2Cl_2 (22.4 mL, 0.26 M) was cooled down to 0 ^oC. The solution was added TEA (3.50 mL, 25.1 mmol) and methanesulfonyl chloride (MsCl) (0.91 mL, 11.7 mmol). After stirring the mixture for 30 min at 0 ^oC and then further at room temperature for 1 h, the reaction mixture was poured into water and extracted with CH_2Cl_2. The combined organic layer was washed with water and dried with $MgSO_4$ and concentrated *in vacuo*. The residue was purified by silica gel flash column chromatography (hexanes:EtOAc=1:1) to afford the title compound as a white solid (1.66 g, 4.62 mmol, 84%). R_f 0.52 (hexanes:EtOAc=1:1).

^{1}H NMR (400 MHz, DMSO-d_6) δ 8.64 (d, J = 8.9 Hz, 1H), 7.47 (d, J = 7.1 Hz, 2H), 7.44–7.33 (m, 5H), 7.21 (t, J = 7.4 Hz, 1H), 7.11 (d, J = 7.9 Hz, 2H), 5.01 (td, J = 8.6, 4.9 Hz, 1H), 4.43–4.29 (m, 2H), 3.22 (s, 3H).

Step 4. Preparation of (*R*)-1-(indolin-5-ylsulfonyl)-4-phenylimidazolidin-2-one (**core 1**): To a suspension of NaH (114 mg, 3.40 mmol) in DMF (17 mL, 0.025 M) was added 1-(2,2,2-trifluoroacetyl)indoline-5-sulfonamide (500 mg, 1.70 mmol) at 0 °C. After stirring the mixture for 10 min at 0 °C, (*R*)-2-((phenoxycarbonyl)amino)-2-phenylethyl methanesulfonate (570 mg, 1.70 mmol) in DMF (17 mL, 0.025 M) was slowly added to the suspension and the resulting mixture was stirred for 3 h at 0 °C and additional 1 h at room temperature. Then NaOH (136 mg, 3.40 mmol) in H_2O (5 mL) was added to the mixture and the resulting solution was stirred for 2 h at room temperature. The reaction mixture was poured into water and the resulting precipitate was filtered with MeOH. The filtrate was dried *in vacuo* to afford the title compound as a white solid (242 mg, 0.705 mmol, two-step 41%). R_f 0.19 (CH_2Cl_2:MeOH=97:3).

^{1}H NMR (300 MHz, DMSO-d_6) δ 8.07 (s, 1H), 7.47 (d, *J* = 7.9 Hz, 2H), 7.40–7.27 (m, 3H), 7.25–7.19 (m, 2H), 6.63 (s, 1H), 6.49 (d, *J* = 8.8 Hz, 1H), 4.75 (dd, *J* = 8.6, 6.1 Hz, 1H), 4.18 (t, *J* = 9.0 Hz, 1H), 3.57 (t, *J* = 8.7 Hz, 2H), 3.39 (dd, *J* = 9.3, 6.1 Hz, 1H), 2.99 (t, *J* = 8.7 Hz, 2H).

(*R*)-1-((1-isonicotinoylindolin-5-yl)sulfonyl)-4-phenylimidazolidin-2-one, (KAS-03)

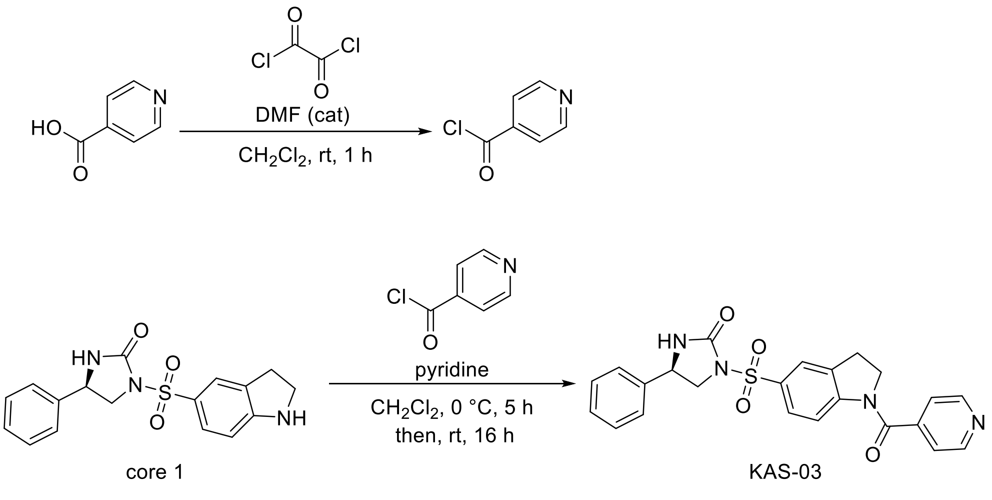

Step 1. Preparation of isonicotinoyl chloride: To a solution of isonicotinic acid (50 mg, 0.406 mmol) was added DMF (1 drop, cat) in CH_2Cl_2 (0.68 mL, 0.6 M). Then oxalyl chloride (0.052 mL, 0.61 mmol) was slowly added to the reaction mixture. After stirring for 1 h at room temperature, the volatiles was removed under vacuum and the crude mixture was co-evaporated twice with CH_2Cl_2. The crude reaction mixture was used without further purification in the next reaction. R_f 0.51 (CH_2Cl_2:MeOH=9:1).

Step 2. Preparation of (*R*)-1-((1-isonicotinoylindolin-5-yl)sulfonyl)-4-phenylimidazolidin-2-one (KAS-03): To a solution of (*R*)-1-(indolin-5-ylsulfonyl)-4-phenylimidazolidin-2-one (**core 1**, 50 mg, 0.146 mmol) in CH_2Cl_2 (1.62 mL, 0.09 M) were added isonicotinoyl chloride (21 mg, 0.146 mmol) and pyridine (0.014 mL, 0.175 mmol) at 0 °C. After stirring the mixture under nitrogen balloon for 5 h at 0 ^{o}C then for 16 h at room temperature, the reaction mixture was poured into water and extracted with CH_2Cl_2. The combined organic layer was washed with water and dried with $MgSO_4$ and concentrated *in vacuo*. The residue was purified by silica gel flash column chromatography (CH_2Cl_2:MeOH=15:1) to afford the title compound as a white solid (6.3 mg, 0.0140 mmol, 10%). R_f 0.23 (CH_2Cl_2:MeOH=9:1)

^{1}H NMR (300 MHz, DMSO-d_6) δ 8.89–8.67 (m, 2H), 8.23 (s, 2H), 7.83 (s, 2H), 7.61 (dd, *J* = 6.0, 2.7 Hz, 2H), 7.43–7.29 (m, 3H), 7.29–7.22 (m, 2H), 4.81 (t, *J* = 7.4 Hz, 1H), 4.29 (t, *J* = 9.0 Hz, 1H), 4.05 (t, *J* = 8.3 Hz, 2H), 3.51 (dd, *J* = 9.4, 6.2 Hz, 1H), 3.18 (t, *J* = 8.4 Hz, 2H).

(*R*)-1-((1-nicotinoylindolin-5-yl)sulfonyl)-4-phenylimidazolidin-2-one, (KAS-05)

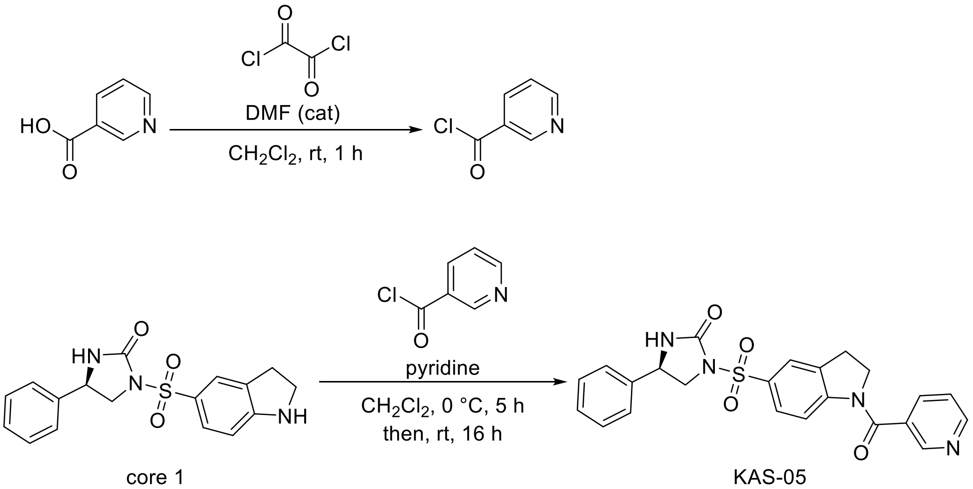

Step 1. Preparation of nicotinoyl chloride: To a solution of nicotinic acid (50 mg, 0.406 mmol) was added DMF (1 drop, cat) in CH_2Cl_2 (0.68 mL, 0.6 M). Then oxalyl chloride (0.052 mL, 0.61 mmol) was slowly added to the reaction mixture. After stirring for 1 h at room temperature, the volatiles was removed under vacuum and the crude mixture was co-evaporated twice with CH_2Cl_2. The crude reaction mixture was used without further purification in the next reaction. R_f 0.51 (CH_2Cl_2:MeOH= 9:1)

Step 2. Preparation of (*R*)-1-((1-nicotinoylindolin-5-yl)sulfonyl)-4-phenylimidazolidin-2-one (KAS-05): To a solution of (*R*)-1-(indolin-5-ylsulfonyl)-4-phenylimidazolidin-2-one (**core 1**, 50 mg, 0.146 mmol) in CH_2Cl_2 (1.62 mL, 0.09 M) were added nicotinoyl chloride (21 mg, 0.146 mmol) and pyridine (0.014 mL, 0.175 mmol) at 0 °C. After stirring the mixture under a nitrogen balloon for 5 h at 0 °C then for 16 h at room temperature, the reaction mixture was poured into water and extracted with CH_2Cl_2. The combined organic layer was washed with water and dried with $MgSO_4$ and concentrated *in vacuo*. The residue was purified by silica gel flash column chromatography (CH_2Cl_2:MeOH=15:1) to afford the title compound as a white

solid (18.7 mg, 0.0417 mmol, 29%). R_f 0.19 (CH_2Cl_2:MeOH=9:1).

^{1}H NMR (300 MHz, Acetonitrile-d_3) δ 8.78 (d, J = 2.2 Hz, 1H), 8.71 (dd, J = 4.9, 1.7 Hz, 1H), 8.06 (br s, 1H), 7.95 (dt, J = 7.9, 2.0 Hz, 1H), 7.82 (s, 1H), 7.80 (d, J = 6.9 Hz, 1H), 7.48 (ddd, J = 8.6, 5.0, 0.9 Hz, 1H), 7.43–7.32 (m, 3H), 7.26–7.22 (m, 2H), 6.15 (s, 1H), 4.79 (t, J = 7.5 Hz, 1H), 4.31 (t, J = 9.1 Hz, 1H), 4.11 (t, J = 8.4 Hz, 2H), 3.60 (dd, J = 9.5, 6.5 Hz, 1H), 3.19 (t, J = 8.4 Hz, 2H).

(*R*)-1-((1-(4-aminobenzoyl)indolin-5-yl)sulfonyl)-4-phenylimidazolidin-2-one, (KAS-01), (*R*)-1-((1-(4-(hydroxyamino)benzoyl)indolin-5-yl)sulfonyl)-4-phenylimidazolidin-2-one, (KAS-07), and (*R*)-1-((1-(4-nitrobenzoyl)indolin-5-yl)sulfonyl)-4-phenylimidazolidin-2-one, (KAS-09)

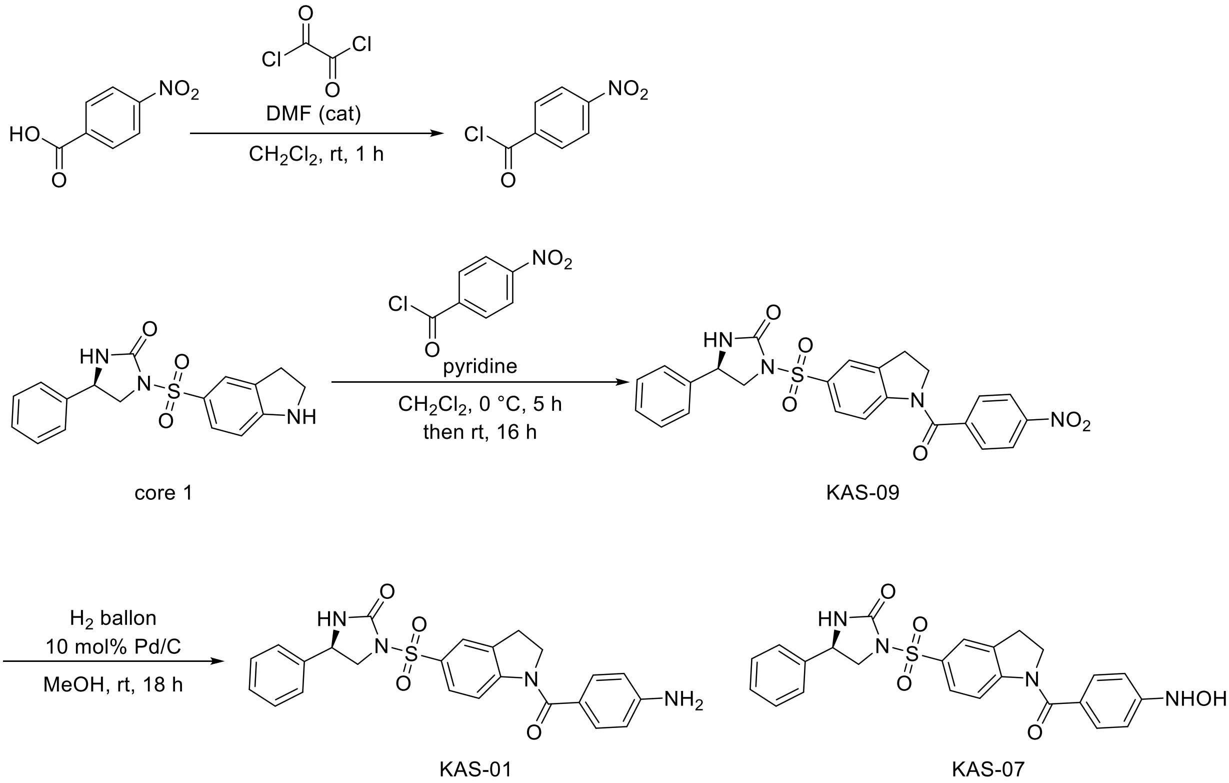

Step 1. Preparation of 4-nitrobenzoyl chloride: To a solution of 4-nitrobenzoic acid (100 mg, 0.600 mmol) was added DMF (1 drop, cat) in CH_2Cl_2 (1.0 mL, 0.6 M). Then oxalyl chloride (0.077 mL, 0.90 mmol) was slowly added to the reaction mixture. After stirring for 1 h at room temperature, the volatiles was removed under vacuum and the crude mixture was co-evaporated twice with CH_2Cl_2. The crude reaction mixture was used without further purification in the next reaction. R_f 0.91 (CH_2Cl_2:MeOH=9:1).

Step 2. Preparation of (*R*)-1-((1-(4-nitrobenzoyl)indolin-5-yl)sulfonyl)-4-phenylimidazolidin-2-one (KAS-09): To a solution of (*R*)-1-(indolin-5-ylsulfonyl)-4-phenylimidazolidin-2-one (**core 1**, 100 mg, 0.292 mmol) in CH_2Cl_2 (3.24 mL, 0.09 M) were added 4-nitrobenzoyl chloride (41 mg, 0.292 mmol) and pyridine (0.118 mL, 1.46 mmol) at 0 °C. After stirring the mixture under a nitrogen balloon for 5 h at 0 °C then for 16 h at room temperature, the reaction mixture was poured into water and extracted with CH_2Cl_2. The combined organic layer was washed with water and dried with $MgSO_4$ and concentrated *in vacuo*. The residue was purified by silica gel flash column chromatography (CH_2Cl_2:MeOH=15:1) to afford the title compound as a yellow solid (32.1 mg, 0.065 mmol, 22%). R_f 0.46 (CH_2Cl_2:MeOH=9:1).

^{1}H NMR (400 MHz, DMSO-d_6) δ 8.36 (d, J = 8.8 Hz, 2H), 8.23 (s, 2H), 7.91 (d, J = 8.7 Hz, 2H), 7.83 (s, 2H), 7.44–7.30 (m, 3H), 7.30–7.17 (m, 2H), 4.81 (t, J = 7.4 Hz, 1H), 4.29 (t, J = 9.0 Hz, 1H), 4.06 (t, J = 8.4 Hz, 2H), 3.52 (dd, J = 9.4, 6.2 Hz, 1H), 3.18 (t, J = 8.4 Hz, 2H).

Step 3. Preparation of (*R*)-1-((1-(4-aminobenzoyl)indolin-5-yl)sulfonyl)-4-phenylimidazolidin-2-one (KAS-01) and (*R*)-1-((1-(4-(hydroxyamino)benzoyl)indolin-5-yl)sulfonyl)-4-phenylimidazolidin-2-one (KAS-07): To a solution of (*R*)-1-((1-(4-nitrobenzoyl)indolin-5-yl)sulfonyl)-4-phenylimidazolidin-2-one (**KAS-09**, 24 mg, 0.048

mmol) in MeOH (4.8 mL, 0.01 M) was added Pd/C (4 mg, 0.0048 mmol). After stirring the mixture for 18 h under an atmosphere of hydrogen (1 atm) using a balloon at room temperature, the mixture was filtered through celite and the crude filtrate was purified by preparative TLCs (CH_2Cl_2:MeOH=9:1) to afford **KAS-01** (R_f 0.44) as a white solid (3.8 mg, 0.0082 mmol, 17%) and **KAS-07** (R_f 0.48) as a white solid (2.1 mg, 0.0044 mmol, 9%).

KAS-01 ^{1}H NMR (400 MHz, DMSO-d_6) δ 8.21 (s, 1H), 7.77 (s, 1H), 7.73 (s, 2H), 7.42–7.30 (m, 5H), 7.28–7.22 (m, 2H), 6.59 (d, J = 8.6 Hz, 2H), 5.77 (s, 2H), 4.80 (t, J = 8.7 Hz, 1H), 4.27 (t, J = 9.0 Hz, 1H), 4.18 (t, J = 8.4 Hz, 2H), 3.50 (dd, J = 9.4, 6.1 Hz, 1H), 3.15 (t, J = 8.3 Hz, 2H).

KAS-07 ^{1}H NMR (300 MHz, DMSO-d_6) δ 8.20 (s, 1H), 7.77 (s, 1H), 7.73 (s, 2H), 7.46 (d, J = 8.6 Hz, 2H), 7.42–7.29 (m, 3H), 7.27–7.17 (m, 2H), 6.58 (d, J = 8.7 Hz, 2H), 6.36 (q, J = 5.1 Hz, 1H), 4.80 (t, J = 7.4 Hz, 1H), 4.27 (t, J = 8.9 Hz, 1H), 4.19 (t, J = 8.4 Hz, 2H), 3.50 (dd, J = 9.4, 6.1 Hz, 1H), 3.15 (t, J = 8.3 Hz, 1H), 2.73 (d, J = 4.9 Hz, 2H).

(*S*)-1-(indolin-5-ylsulfonyl)-4-phenylimidazolidin-2-one, (core 2)

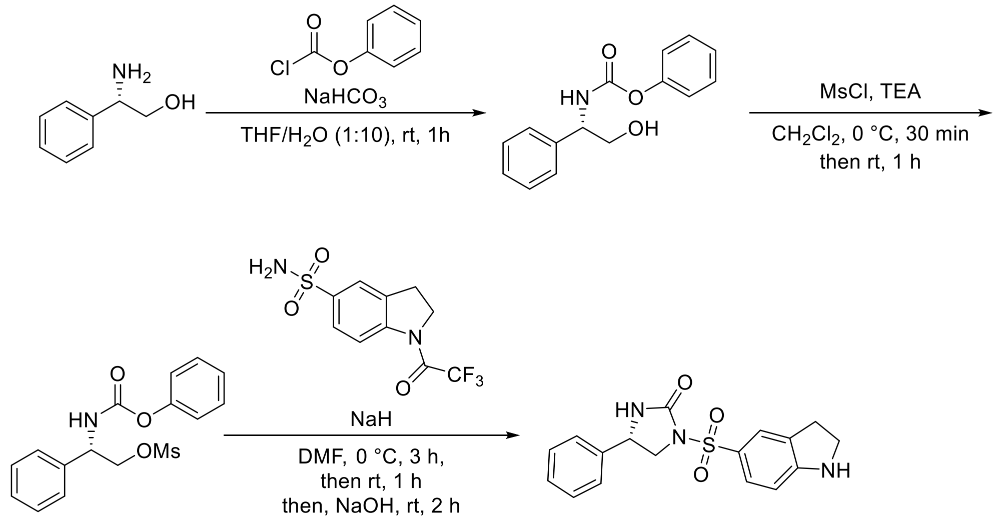

core 2

Step 1. Preparation of phenyl (*S*)-(2-hydroxy-1-phenylethyl)carbamate: To a solution of (*S*)-(+)-2-phenylglycinol (1.50 g, 10.9 mmol) and sodium bicarbonate (1.38 g, 16.4 mmol) in H_2O (30 mL) was slowly added phenylchloroformate (1.50 mL, 12.0 mmol) in THF (3 mL). After stirring the mixture for 1 h at room temperature, the reaction mixture was poured into water and extracted with EtOAc. The combined organic layer was washed with water, dried with $MgSO_4$, and concentrated *in vacuo* to afford the compound as a white solid (1.93 g, 7.501 mmol, 69%). R_f 0.5 (CH_2Cl_2:MeOH=19:1).

^{1}H NMR (300 MHz, DMSO-d_6) δ 8.23 (d, *J* = 8.6 Hz, 1H), 7.40–7.31 (m, 6H), 7.30–7.25 (m, 1H), 7.19 (t, *J* = 7.4 Hz, 1H), 7.09 (d, *J* = 7.9 Hz, 2H), 4.95 (t, *J* = 5.7 Hz, 1H), 4.63 (q, *J* = 7.2 Hz, 1H), 3.59 (t, *J* = 6.2 Hz, 2H).

Step 2. **Preparation of (*S*)-2-((phenoxycarbonyl)amino)-2-phenylethylmethanesulfonate**: To a solution of (*S*)-(2-hydroxy-1-phenylethyl)carbamate (1.50 g, 5.83 mmol) in CH_2Cl_2 (22.4 mL, 0.26 M) were added TEA (3.50 mL, 25.1 mmol) and MsCl (0.91 mL, 11.7 mmol) at 0 °C. After stirring the mixture for 30 min at 0 °C and then further at room temperature for 1 h, the reaction mixture was poured into water, dried with $MgSO_4$, and concentrated *in vacuo*. The residue was purified by silica gel flash column chromatography (hexanes:EtOAc=1:1) to afford the compound as a white solid (1.39 g, 4.145 mmol, 71%). R_f 0.86 (CH_2Cl_2:MeOH=19:1).

^{1}H NMR (300 MHz, DMSO-d_6) δ 8.63 (d, *J* = 8.9 Hz, 1H), 7.47 (d, *J* = 7.1 Hz, 2H), 7.45–7.33 (m, 5H), 7.22 (t, *J* = 7.4 Hz, 1H), 7.11 (d, *J* = 7.4 Hz, 2H), 5.01 (td, *J* = 8.5, 5.2 Hz, 1H), 4.45–4.31 (m, 2H), 3.22 (s, 3H).

Step 3. Preparation of (*S*)-1-(indolin-5-ylsulfonyl)-4-phenylimidazolidin-2-one (core 2): To a solution of 1-(2,2,2-trifluoroacetyl)indoline-5-sulfonamide (400 mg, 1.36 mmol) in

anhydrous DMF (13.6 mL, 0.025 M) was added NaH (65 mg, 2.72 mmol) at 0 °C. After stirring the mixture for 10 min at 0 °C, (*S*)-2-((phenoxycarbonyl)amino)-2-phenylethyl methanesulfonate (456 mg, 1.36 mmol) in DMF (13.6 mL, 0.025 M) was slowly added to the suspension and the resulting mixture was stirred for 3 h at 0 °C and additional 1 h at room temperature. Then NaOH (54 mg, 1.36 mmol) in H_2O (10 mL) was added to the mixture and the resulting solution was stirred for 2 h at room temperature. The reaction mixture was poured into water and the resulting precipitate was filtered with MeOH. The filtrate was dried *in vacuo* to afford the title compound as a white (171 mg, 0.498 mmol, two-step 37%). R_f 0.25 (CH_2Cl_2:MeOH=97:3).

^{1}H NMR (300 MHz, DMSO-d_6) δ 8.07 (s, 1H), 7.47 (d, *J* = 7.9 Hz, 2H), 7.40–7.30 (m, 3H), 7.25–7.20 (m, 2H), 6.64 (s, 1H), 6.50 (d, *J* = 8.1 Hz, 1H), 4.75 (dd, *J* = 8.5, 6.4 Hz, 1H), 4.18 (t, *J* = 9.0 Hz, 1H), 3.58 (t, *J* = 8.7 Hz, 2H), 3.40 (dd, *J* = 9.3, 6.1 Hz, 1H), 3.00 (t, *J* = 8.8 Hz, 2H).

(*S*)-1-((1-isonicotinoylindoli-5-yl)sulfonyl)-4-phenylimidazolidin-2-one, (KAS-02)

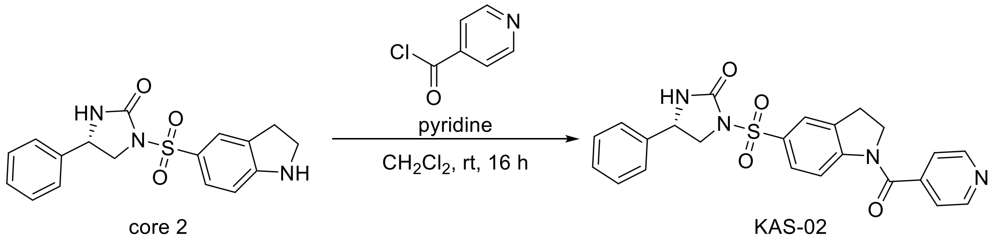

Step 1. Preparation of (*S*)-1-((1-isonicotinoylindoli-5-yl)sulfonyl)-4-phenylimidazolidin-2-one (KAS-02): To a solution of (*S*)-1-(indolin-5-ylsulfonyl)-4-phenylimidazolidin-2-one (**core 2**, 100 mg, 0.291 mmol) in CH_2Cl_2 (3.23 mL, 0.09 M) were added isonicotinyl chloride (41 mg, 0.291 mmol) and pyridine (0.117 mL, 1.455 mmol). After stirring the mixture under

argon balloon for 16 h at room temperature, the reaction mixture was poured into water and extracted with CH_2Cl_2. The combined organic layer was washed with water, dried with $MgSO_4$, and concentrated *in vacuo*. The residue was purified by preparatory TLCs (CH_2Cl_2:MeOH=9:1) to afford the compound as a white solid (62 mg, 0.138 mmol, 48%). R_f 0.3 (CH_2Cl_2:MeOH=9:1).

^{1}H NMR (300 MHz, DMSO-d_6) δ 8.80–8.74 (m, 2H), 8.24 (s, 2H), 7.84 (s, 2H), 7.62 (dd, J = 4.4, 2.8 Hz, 2H), 7.46–7.30 (m, 3H), 7.30–7.22 (m, 2H), 4.82 (t, J = 7.4 Hz, 1H), 4.30 (t, J = 9.1 Hz, 1H), 4.06 (t, J = 8.4 Hz, 2H), 3.52 (dd, J = 9.6, 6.2 Hz, 1H), 3.19 (t, J = 8.4 Hz, 2H).

(*S*)-1-((1-nicotinoylindoli-5-yl)sulfonyl)-4-phenylimidazolidin-2-one, (KAS-04)

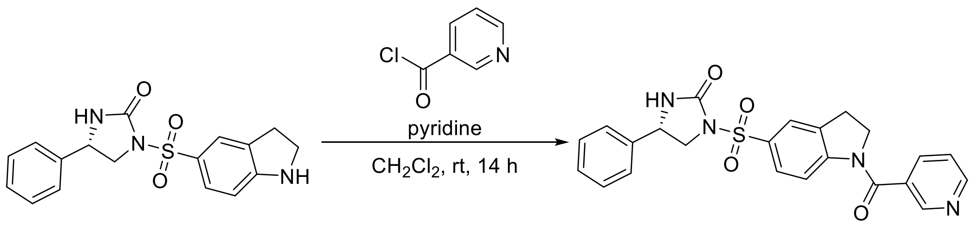

Step 1. Preparation of (*S*)-1-((1-nicotinoylindolin-5-yl)sulfonyl-4-phenylimidazolidin-2-one (KAS-04): To a solution of (*S*)-1-(indolin-5-ylsulfonyl)-4-phenylimidazolidin-2-one (**core2,** 30 mg, 0.087 mmol) in CH_2Cl_2 (0.97 mL, 0.09 M) were added nicotinoyl chloride (12 mg, 0.087 mmol) and pyridine (0.035 mL, 0.435 mmol). After stirring the mixture under an argon balloon for 14 h at room temperature, the resulting solution was poured into water and extracted with CH_2Cl_2. The combined organic layer was washed with water, dried with $MgSO_4$, and concentrated *in vacuo*. The residue was purified by preparatory TLCs (CH_2Cl_2:MeOH=9:1) to afford the compound as a white solid (15 mg, 0.0334 mmol, 38%). R_f 0.55 (CH_2Cl_2:MeOH=9:1).

¹H NMR (300 MHz, Acetonitrile-d_3) δ 8.81 (d, *J* = 1.4 Hz, 1H), 8.74 (dd, *J* = 4.9, 1.7 Hz, 1H), 8.06 (br s, 1H), 7.98 (dt, *J* = 7.9, 2.0 Hz, 1H), 7.85 (s, 1H), 7.83 (d, *J* = 7.1 Hz, 1H), 7.51 (ddd, *J* = 7.9, 4.9, 0.9 Hz, 1H), 7.45–7.35 (m, 3H), 7.31–7.23 (m, 2H), 6.32 (s, 1H), 4.81 (t, *J* = 7.5 Hz, 1H), 4.33 (t, *J* = 9.1 Hz, 1H), 4.14 (t, *J* = 8.4 Hz, 2H), 3.62 (dd, *J* = 9.5, 6.5 Hz, 1H), 3.22 (t, *J* = 8.4 Hz, 2H).

(*S*)-1-((1-4-aminobenzoyl)indolin-5-yl)sulfonyl)-4-phenylimidazolidin-2-one, (DW2282), (*S*)-1-((1-4-hydroxyaminobenzoyl)indolin-5-yl)sulfonyl)-4-phenylimidazolidin-2-one, (KAS-06), and (*S*)-1-((1-4-nitrobenzoyl)indolin-5-yl)sulfonyl)-4-phenylimidazolidin-2-one, (KAS-08)

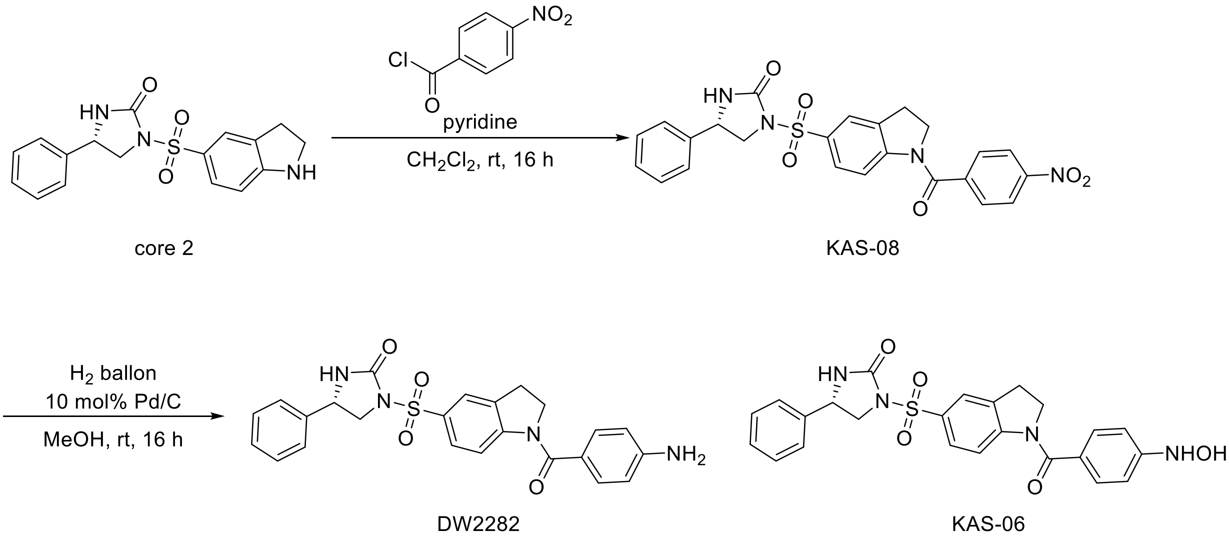

Step 1. Preparation of (*S*)-1-((1-4-nitrobenzoyl)indolin-5-yl)sulfonyl)-4-phenylimidazolidin-2-one (KAS-08): To a solution of (*S*)-1-(indolin-5-ylsulfonyl)-4-phenylimidazolidin-2-one (**core 2,** 150 mg, 0.437 mmol) in CH_2Cl_2 (4.85 mL, 0.09 M) were added 4-nitrobenzoyl chloride (79 mg, 0.437 mmol) and pyridine (0.174 mL, 2.184 mmol). After stirring the mixture under an argon balloon for 16 h at room temperature, the reaction

mixture was poured into water and extracted with CH_2Cl_2. The combined organic layer was washed with water, dried with $MgSO_4$, and concentrated *in vacuo*. The residue was purified by preparatory TLCs (CH_2Cl_2:MeOH=9:1) to afford the compound as a white solid (88 mg, 0.179 mmol, 41%). R_f 0.64 (CH_2Cl_2:MeOH=9:1).

^{1}H NMR (300 MHz, DMSO-d_6) δ 8.37 (d, J = 8.8 Hz, 2H), 8.24 (s, 2H), 7.92 (d, J = 8.7 Hz, 2H), 7.84 (s, 2H), 7.43–7.30 (m, 3H), 7.28–7.24 (m, 2H), 4.82 (t, J = 7.6 Hz, 1H), 4.30 (t, J = 9.0 Hz, 1H), 4.07 (t, J = 8.4 Hz, 2H), 3.52 (dd, J = 9.5, 6.2 Hz, 1H), 3.19 (t, J = 8.3 Hz, 2H).

^{13}C NMR (101 MHz, DMSO-d_6) δ 167.54, 154.98, 148.80, 147.44, 142.70, 141.18, 134.77, 132.70, 129.24, 128.93, 128.55, 128.46, 126.34, 125.00, 124.38, 116.64, 52.76, 51.94, 51.18, 27.83.

HRMS (FAB$^+$) calcd for $C_{24}H_{21}N_4O_6S_1^+$ $[M+H]^+$: 493.1104, found 493.1167

Step 2. Preparation of (*S*)-1-((1-(4-aminobenzoyl)indolin-5-yl)sulfonyl)-4-phenylimidazolidin-2-one (DW2282) and (*S*)-1-((1-(4-hydroxyaminobenzoyl)indolin-5-yl)sulfonyl)-4-phenylimidazolidin-2-one (KAS-06) : To a solution of (*S*)-1-((1-4-nitrobenzoyl)indolin-5-yl)sulfonyl)-4-phenylimidazolidin-2-one (**KAS-08**, 20 mg, 0.041 mmol) in MeOH (4.1 mL, 0.01 M) was added Pd/C (0.43 mg, 0.0041 mmol) and the mixture was stirred at room temperature under hydrogen atmosphere for 16 h at room temperature, then the mixture was filtered through celite and the crude reaction mixture was purified by preparatory TLCs (CH_2Cl_2:MeOH=9:1) to afford **DW2282** (R_f 0.4) as a light purple solid (16 mg, 0.0346 mmol, 84%) and **KAS-06** (R_f 0.45) as a light purple solid (3 mg, 0.00627 mmol, 15%).

DW2282 ^{1}H NMR (300 MHz, DMSO-d_6) δ 8.23 (s, 1H), 7.78 (s, 1H), 7.74 (s, 2H), 7.44–7.29 (m, 5H), 7.28–7.23 (m, 2H), 6.60 (d, *J* = 8.6 Hz, 2H), 5.79 (s, 2H), 4.80 (t, *J* = 8.4 Hz, 1H), 4.28 (t, *J* = 9.1 Hz, 1H), 4.19 (t, *J* = 8.3 Hz, 2H), 3.51 (dd, *J* = 9.4, 6.2 Hz, 1H), 3.15 (t, *J* = 8.3 Hz, 2H).

KAS-06 ^{1}H NMR (300 MHz, DMSO-d_6) δ 8.22 (s, 1H), 7.78 (s, 1H), 7.74 (s, 2H), 7.47 (d, *J* = 8.6 Hz, 2H), 7.43–7.29 (m, 3H), 7.28–7.16 (m, 2H), 6.58 (d, *J* = 8.7 Hz, 2H), 6.38 (q, *J* = 5.0 Hz, 1H), 4.81 (t, *J* = 7.4 Hz,1H), 4.28 (t, *J* = 9.0 Hz, 1H), 4.20 (t, *J* = 8.4 Hz, 2H), 3.50 (dd, *J* = 9.5, 6.1 Hz, 1H), 3.13 (d, *J* = 9.7 Hz, 1H), 2.74 (d, *J* = 5.0 Hz, 2H).

2. Preliminary screening results

Table S1. Structure-activity relationships of 1-(indolin-5-ylsulfonyl)-4-phenylimidazolidin-2-ones[1]

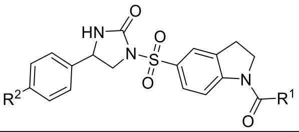

Entry	Compound	R^1	R^2	EC_{50} (μM)	
				WT	STING KO
1	KAS-S01	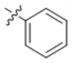	H	1.363	n.d.
2	KAS-S02	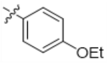	H	n.d.	n.d.
3	KAS-S03	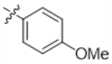	H	0.376	n.d.
4	KAS-S04	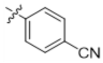	H	0.819	n.d.
5	KAS-S05	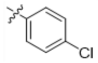	H	0.661	n.d.
6	KAS-S06	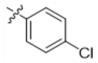	Cl	27.164	n.d.
7	KAS-S07	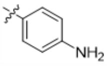	H	0.207	n.d.
8	KAS-S08	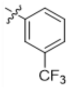	H	0.74	n.d.
9	KAS-S09	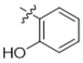	H	0.678	n.d.
10	KAS-S10	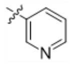	H	0.719	n.d.
11	KAS-S11	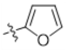	H	0.193	n.d.
12	KAS-S12	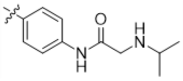	H	0.794	n.d.
13	KAS-S13	CH_3	H	6.188	n.d.
14	KAS-S14	CF_3	H	n.d.	n.d.
15	KAS-S15	$NHCH_2CH_3$	H	0.782	n.d.

16	KAS-S16	-ξ-$NHCH_2CH_3$	Cl	3.594	n.d.
17	KAS-S17	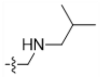	H.	n.d	n.d.

[1] Activity was measured by ISG luciferase reporter assay in THP-1 cells. Individual compound was pre-treated in prior to cGAMP (1 µg/ml), then stimulated for 24 hr. Luciferase signal was normalized by DMSO treatment. EC_{50} and E_{max} was calculated by Logistic regression. EC_{50} > 10 µM means no significant result within the dose range we tested (10-0.04 µM). n.d means not determined.

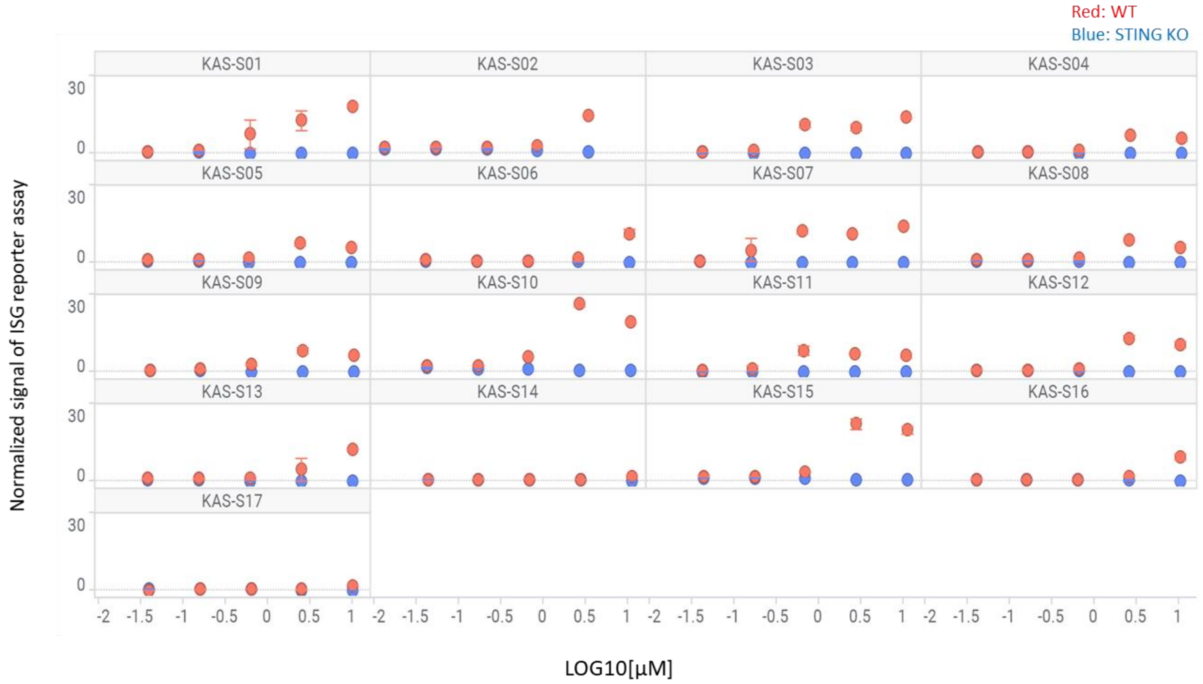

Figure S1. Dose-response graph for Table S1. Luciferase signal was normalized by DMSO control. Graphs show mean and SD.

Table S2. Structure-activity relationships of 1-(indolin-5-ylsulfonyl)-4-phenylpyrrolidin-2-ones

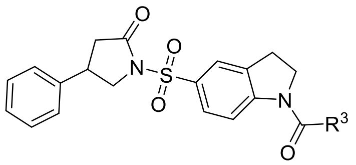

Entry	Compound	R^3	EC_{50} (μM)	
			WT	STING KO
1	KAS-S18	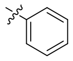	n.d.	n.d.
2	KAS-S19	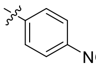	n.d.	n.d.
3	KAS-S20	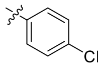	n.d.	n.d.
4	KAS-S21	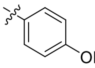	n.d.	n.d.
5	KAS-S22	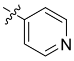	n.d.	n.d.
6	KAS-S23	-§-CH_3	n.d.	n.d.
7	KAS-S24	-§-CF_3	n.d.	n.d.

[1] Activity was measured by ISG luciferase reporter assay in THP-1 cells. Individual compound was pre-treated in prior to cGAMP (1 μg/ml), then stimulated for 24 hr. Luciferase signal was normalized by DMSO treatment. EC_{50} and E_{max} was calculated by Logistic regression. EC_{50} > 10 μM means no significant result within the dose range we tested (10-0.04 μM). n.d means not determined.

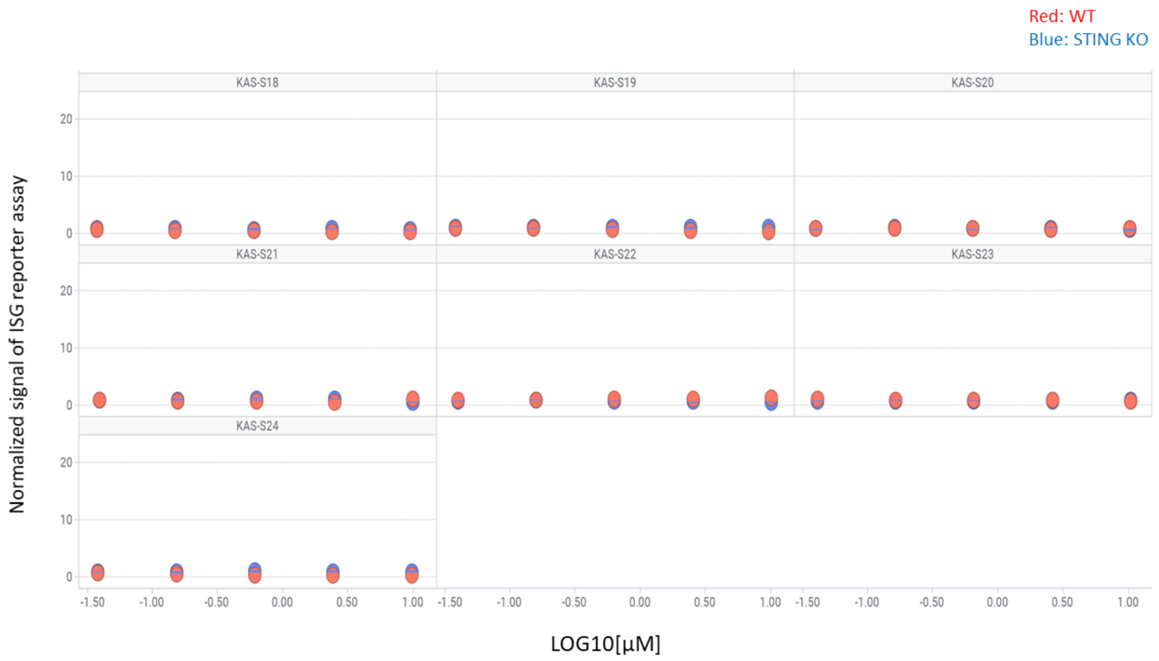

Figure S2. Dose-response graph for Table S2. Luciferase signal was normalized by DMSO control. Graphs show mean and SD.

3. Dose-response graphs for Table 1

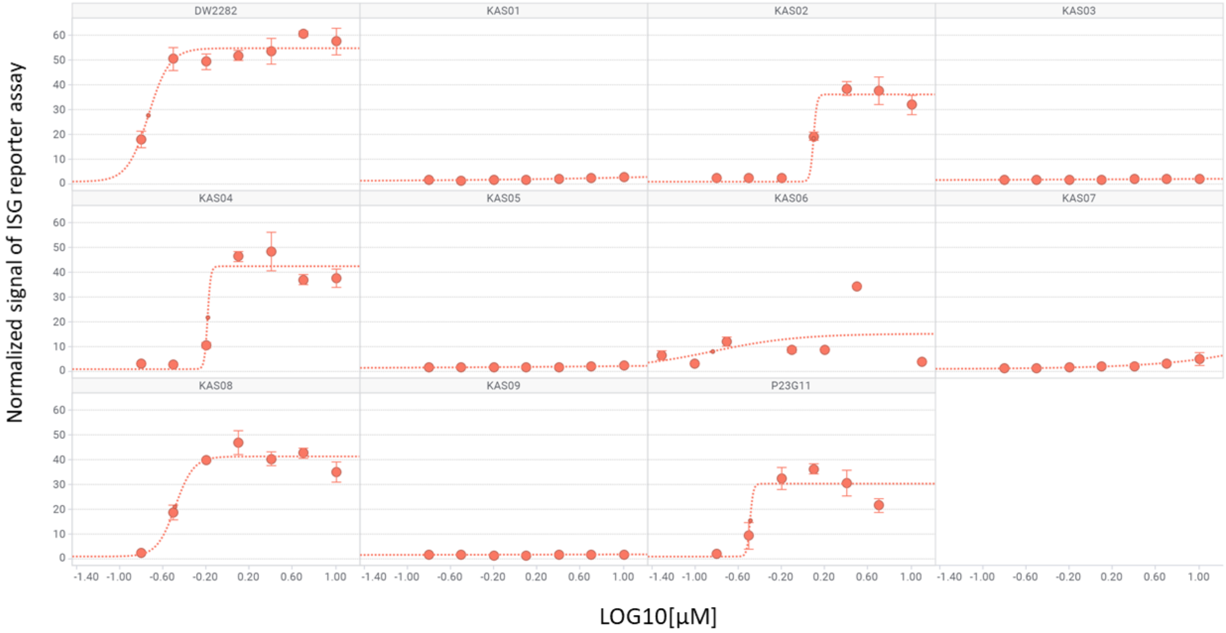

Figure S3. Result for ISG reporter assay in Table 1. Luciferase signal was normalized by DMSO control. Graphs show mean and SD.

4. Cell viability test for KAS-08

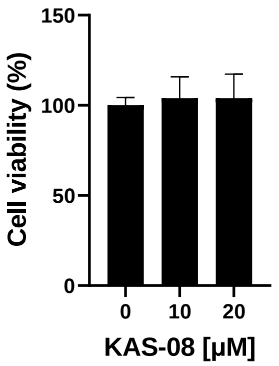

Figure S4. Result for cell viability of KAS-08 in PBMC. PBMC were obtained from Zenbio. Indicated concentration of KAS-08 was treated for 24 hr, then monitored by cytotoxic assay (Promega, CellTiter Glo Assay). Graph shows mean and SD.

5. ^{1}H NMR spectrums

(*S*)-1-((1-4-aminobenzoyl)indolin-5-yl)sulfonyl)-4-phenylimidazolidin-2-one, (DW2282)

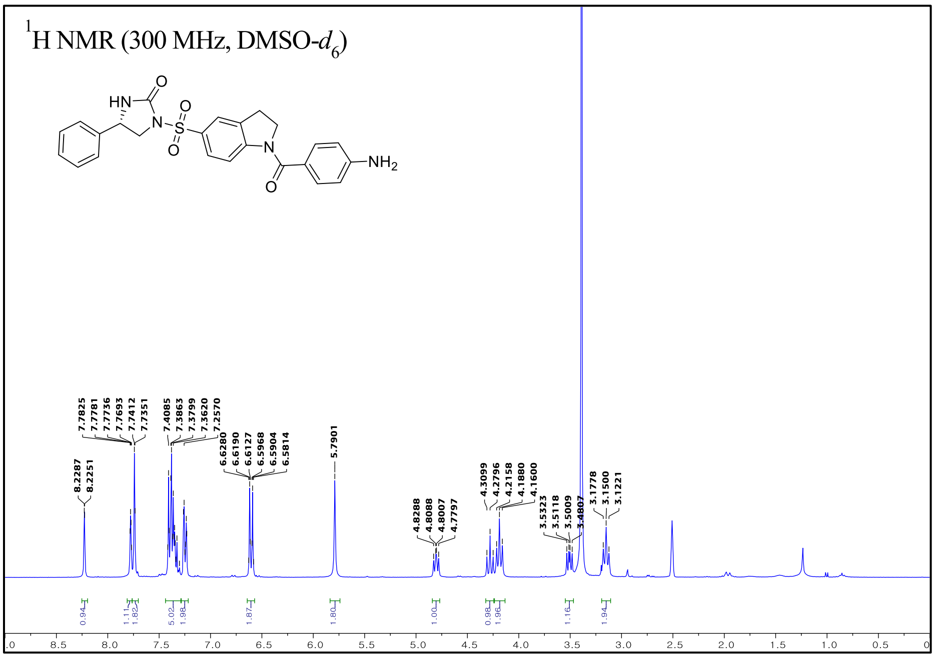

(*R*)-1-((1-(4-aminobenzoyl)indolin-5-yl)sulfonyl)-4-phenylimidazolidin-2-one, (KAS-01)

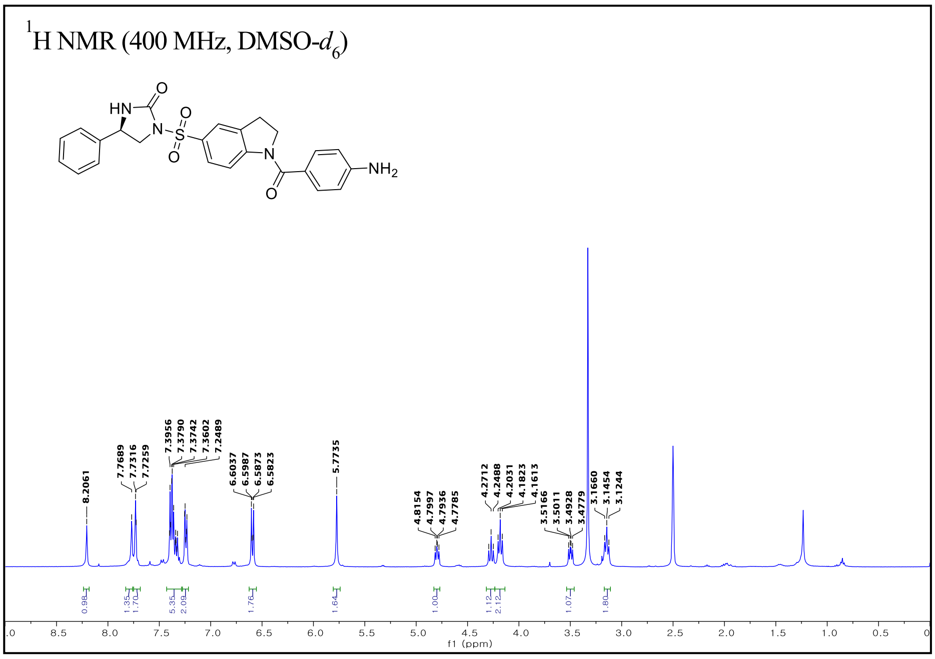

(*S*)-1-((1-isonicotinoylindoli-5-yl)sulfonyl)-4-phenylimidazolidin-2-one, (KAS-02)

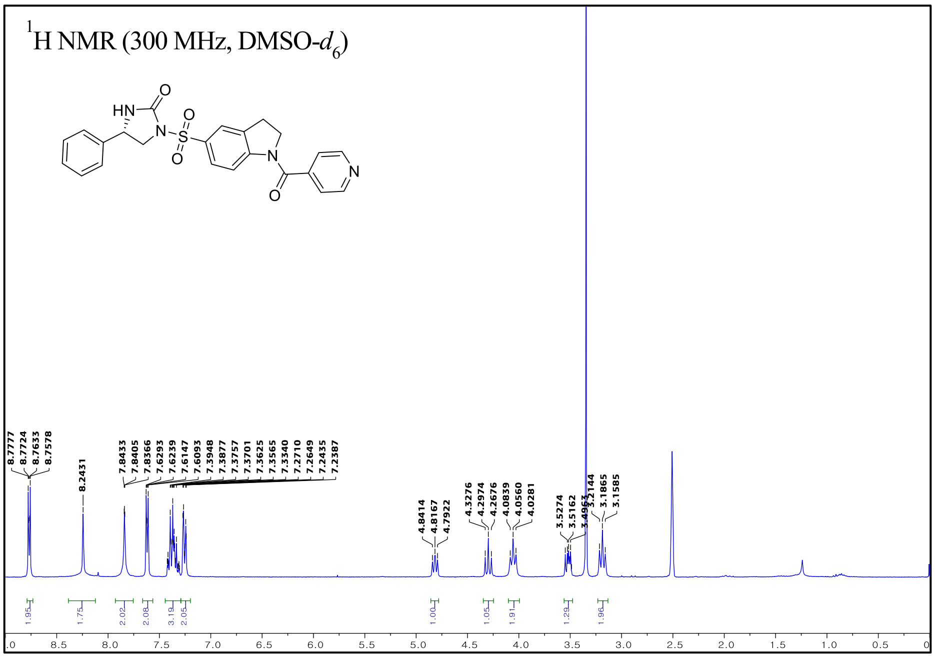

(*R*)-1-((1-isonicotinoylindolin-5-yl)sulfonyl)-4-phenylimidazolidin-2-one, (KAS-03)

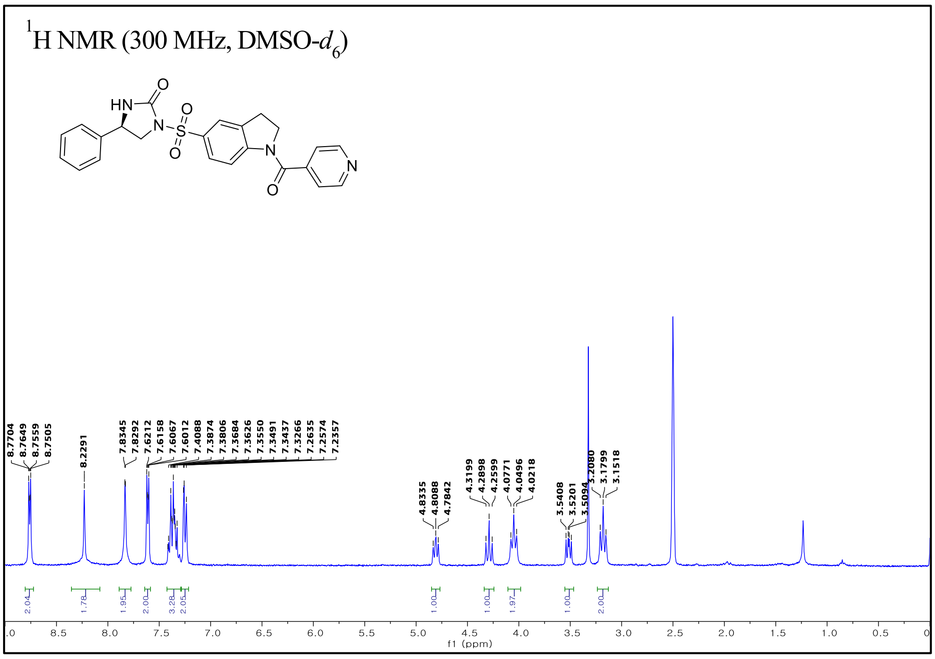

(*S*)-1-((1-nicotinoylindoli-5-yl)sulfonyl)-4-phenylimidazolidin-2-one, (KAS-04)

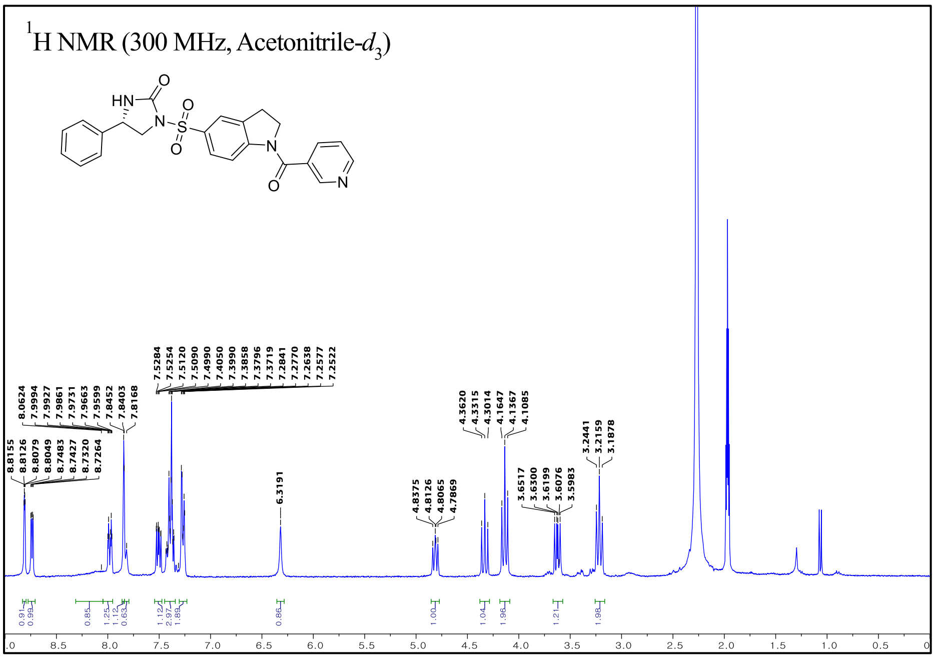

(*R*)-1-((1-nicotinoylindolin-5-yl)sulfonyl)-4-phenylimidazolidin-2-one, (KAS-05)

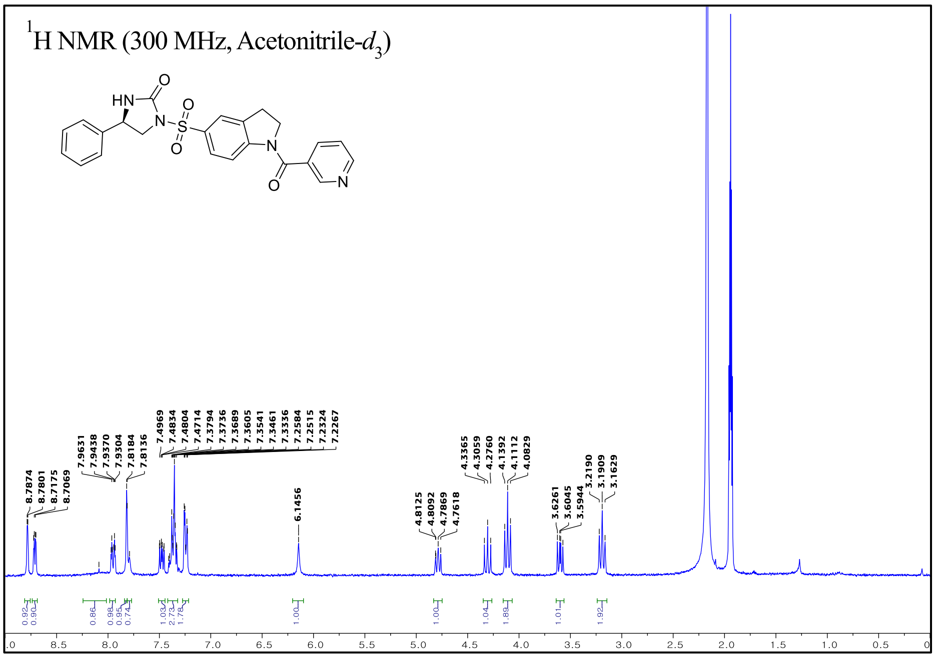

(*S*)-1-((1-4-hydroxyaminobenzoyl)indolin-5-yl)sulfonyl)-4-phenylimidazolidin-2-one, (KAS-06)

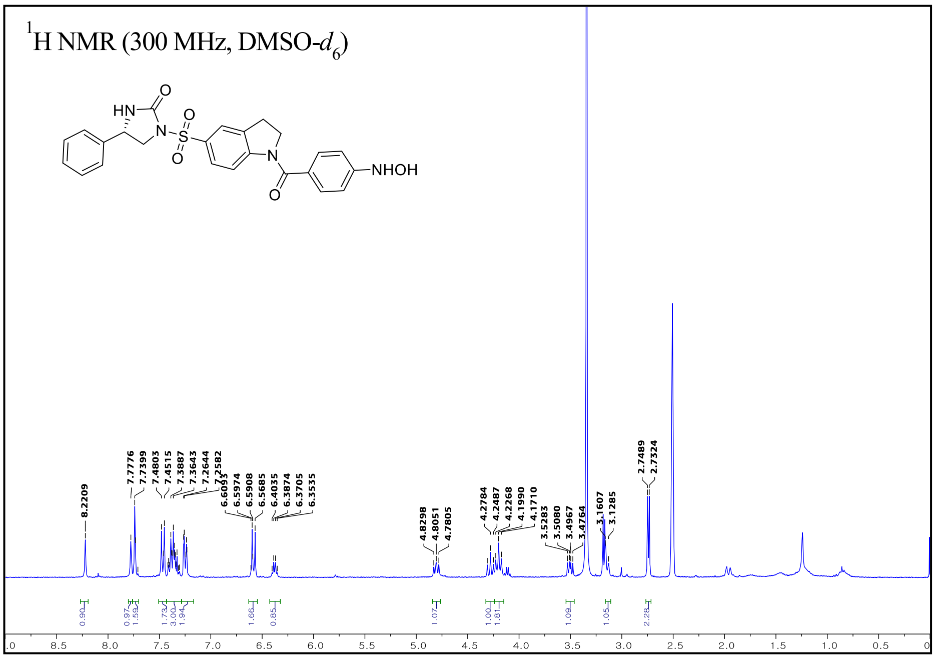

(*R*)-1-((1-(4-(hydroxyamino)benzoyl)indolin-5-yl)sulfonyl)-4-phenylimidazolidin-2-one, (KAS-07)

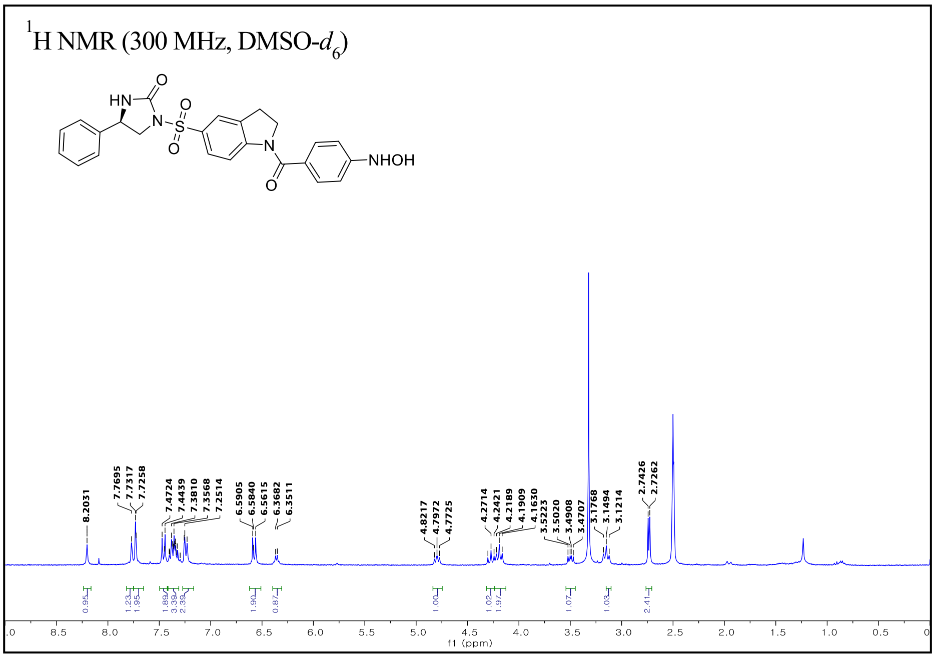

(*S*)-1-((1-4-nitrobenzoyl)indolin-5-yl)sulfonyl)-4-phenylimidazolidin-2-one, (KAS-08)

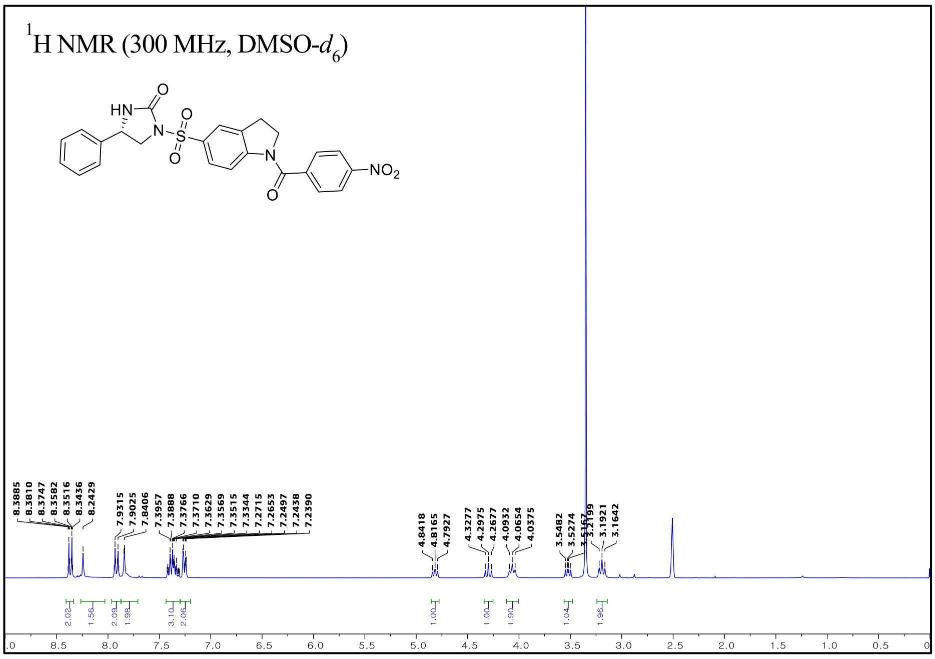

(*R*)-1-((1-(4-nitrobenzoyl)indolin-5-yl)sulfonyl)-4-phenylimidazolidin-2-one, (KAS-09)

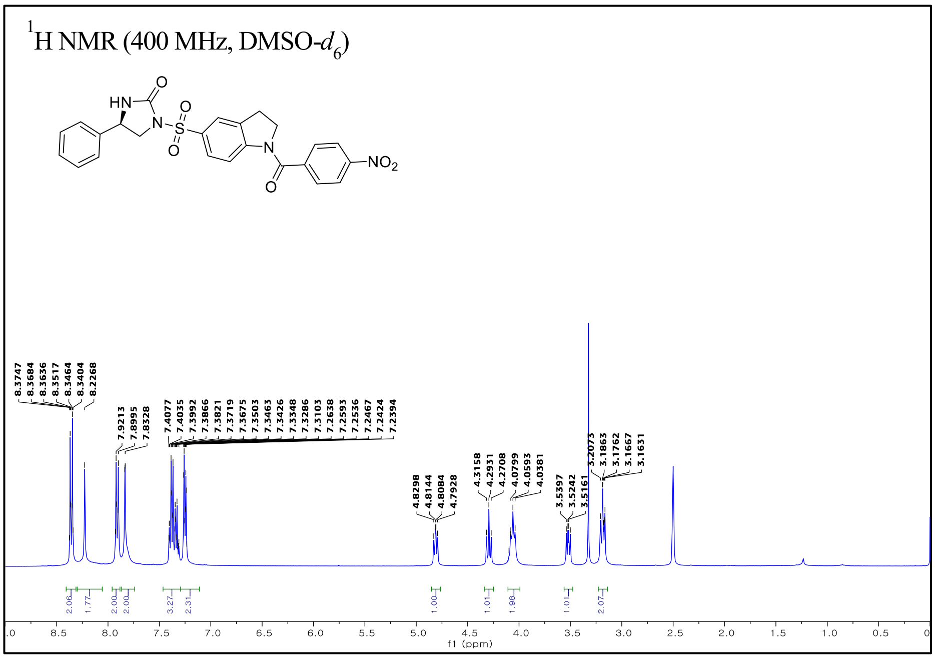

Supplement: Supplementary file 1 [file biomedicines-10-00033-s001.zip › biomedicines-1504264-supplementary.pdf]
